# Liver-specific adiponectin gene therapy suppresses microglial NLRP3-inflammasome activation for treating Alzheimer’s disease

**DOI:** 10.1186/s12974-024-03066-y

**Published:** 2024-03-27

**Authors:** Roy Chun-Laam Ng, Min Jian, Oscar Ka-Fai Ma, Ariya Weiman Xiang, Myriam Bunting, Jason Shing-Cheong Kwan, Curtis Wai-Kin Wong, Leung-Wah Yick, Sookja Kim Chung, Karen Siu-Ling Lam, Ian E. Alexander, Aimin Xu, Koon-Ho Chan

**Affiliations:** 1 Department of Medicine, LKS Faculty of Medicine, The University of Hong Kong, Queen Mary Hospital, 4/F, Professorial Block, 102 Pokfulam Road, Hong Kong, Special Administrative Region China; 2 Neuroimmunology and Neuroinflammation Research Laboratory, LKS Faculty of Medicine, The University of Hong Kong, Hong Kong, China; 3 Division of Neuroscience, Faculty of Biology, Medicine and Health, School of Biological Sciences, University of Manchester, Manchester, UK; 4 Faculty of Medicine, Dr. Neher’s Biophysics Laboratory for Innovative Drug Discovery at Macau University of Science and Technology, Taipa, Macao, China; 5 Research Center of Heart, Brain, Hormone and Healthy Aging, The University of Hong Kong, Pokfulam, Hong Kong, China; 6 Gene Therapy Research Unit, Faculty of Medicine and Health, Children’s Medical Research Institute and Sydney Children’s Hospitals Network, The University of Sydney, Westmead, NSW Australia

**Keywords:** Gene delivery, Alzheimer’s Disease, Adiponectin, NLRP3, Neuroinflammation

## Abstract

Adiponectin (APN) is an adipokine which predominantly expresses in adipocytes with neuroprotective and anti-inflammatory effects. We have recently indicated that circulatory trimeric APN can enter the brain by crossing the blood–brain barrier (BBB) and modulate microglia-mediated neuroinflammation. Here, we found that the microglial NLR family pyrin domain containing 3 (NLRP3)-inflammasome activation was exacerbated in *APN*^*−/−*^5xFAD mice in age-dependent manner. The focus of this study was to develop a new and tractable therapeutic approach for treating Alzheimer’s disease (AD)-related pathology in 5xFAD mice using peripheral APN gene therapy. We have generated and transduced adeno-associated virus (AAV2/8) expressing the mouse mutated *APN* gene (APN^C39S^) into the liver of 5xFAD mice that generated only low-molecular-weight trimeric APN (APN^Tri^). Single dose of AAV2/8-APN^C39S^ in the liver increased circulatory and cerebral APN levels indicating the overexpressed APN^Tri^ was able to cross the BBB. Overexpression of APN^Tri^ decreased both the soluble and fibrillar Aβ in the brains of 5xFAD mice. AAV2/8-APN^Tri^ treatment reduced Aβ-induced IL-1β and IL-18 secretion by suppressing microglial NLRP3-inflammasome activation. The memory functions improved significantly in AAV-APN^Tri^-treated 5xFAD mice with reduction of dystrophic neurites. These findings demonstrate that peripheral gene delivery to overexpress trimeric APN can be a potential therapy for AD.

**Graphical Abstract:**

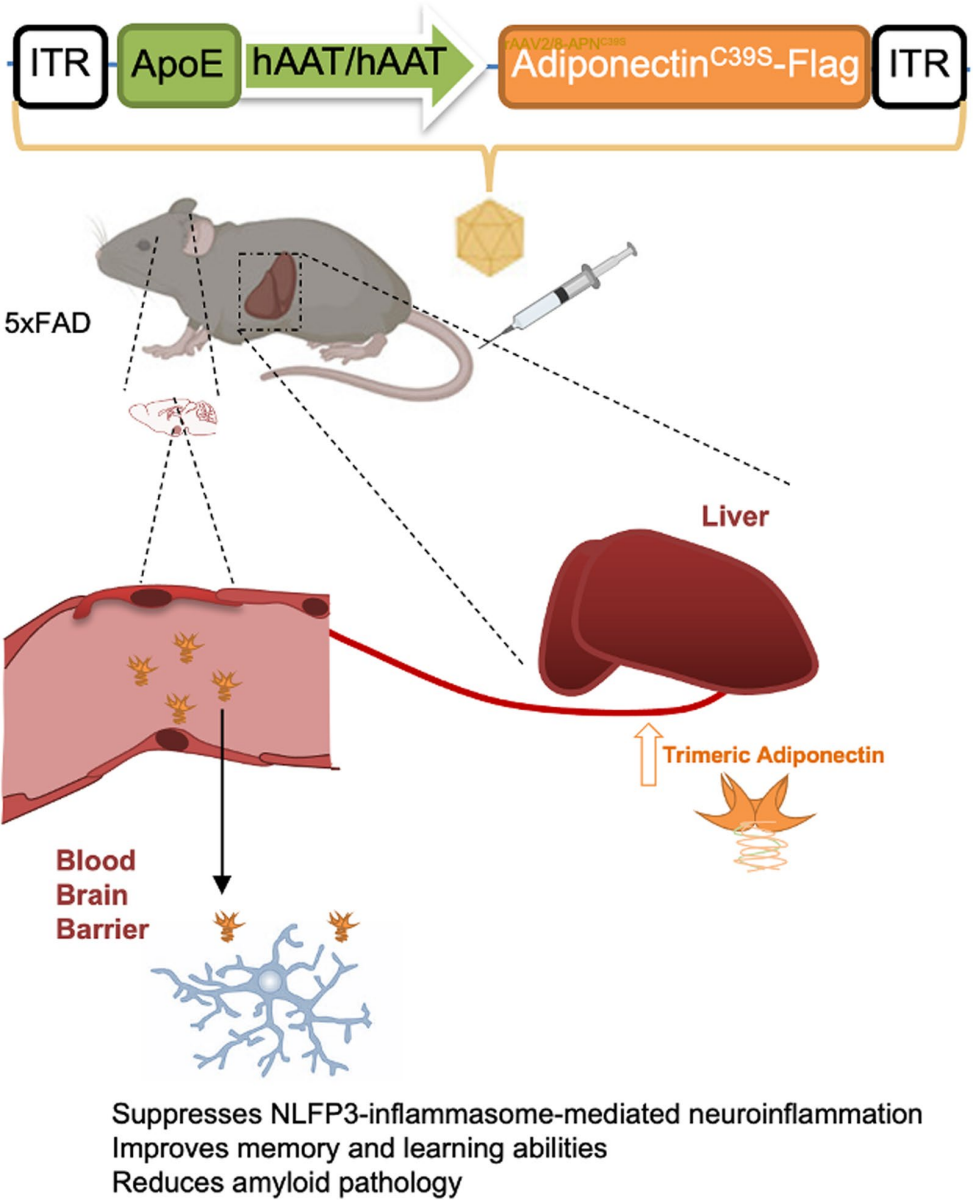

**Supplementary Information:**

The online version contains supplementary material available at 10.1186/s12974-024-03066-y.

## Introduction

Alzheimer’s disease (AD) is the most prevalent neurodegenerative disorder and is characterized by the progressive loss of neurons in regions of the cerebrum (the neocortex and hippocampus) that are related to memory and higher cortical function. Most cases of AD are sporadic in which the risk of developing AD depends on the interactions between environmental, genetic, lifestyle, and epigenetic factors. The pathogenesis of AD is associated with the formation of amyloid plaques and neurofibrillary tangles (NFTs), which result from the accumulation of extracellular Aβ and the intracellular deposition of hyperphosphorylated tau proteins, respectively [1]. However, other pathogenic factors are also involved in the etiology of AD.

Recent studies have indicated that neuroinflammation plays a central role in the pathophysiology and progression of AD, with microglia as the key cell of the inflammatory response [2]. The aggregation of Aβ could activate a proinflammatory downstream cascade of microglia that leads to and exacerbates AD pathology. Numerous inflammatory markers have been found in the AD brain, including increased levels of inflammatory cytokines. and chemokines, as well as the accumulation of activated microglia in damaged areas [3]. Transcriptomic and epigenomic analyses of human microglia revealed diversity of microglial states (including homeostatic, inflammatory, lipid processing and phagocytosing) across disease with disease-stage changes of gene expression [4]. Increasing evidence supports that the NLRP3 inflammasome plays a crucial role in the pathogenesis of AD [5–7]. Furthermore, soluble Aβ oligomers (AβO) and fibrillar Aβ can trigger activation of the NLRP3 inflammasome in the microglia, which leads to the maturation of pro-IL-1β and generation of IL-1β [8]. Extracellular Apoptosis-associated speck like protein containing a CARD (ASC) binds to Aβ to form Aβ-ASC complex which can further enhance amyloid seeding and activate NLRP3 inflammasome [9]. Aβ aggregates also enhance the translocation of NF-kB to the nucleus, initiating the transcription of NLRP3, ASC, pro-caspase-1, pro-IL-1β. Deletion of NLRP3 in AD mice could reduce Aβ-mediate neuroinflammation and cognitive impairments [5]. Following with these important findings, more articles have demonstrated that inhibition of the NLRP3 inflammasome pathway enhanced the clearance of Aβ by upregulating the levels of neprilysin or by shifting microglia to the anti-inflammatory [10, 11] M2 phenotype in AD models. Therefore, an agent which can inhibit NLRP3-inflammasome activation may a potential treatment for AD.

Our group has reported that adiponectin (APN) can modulate microglial activities including phagocytosis and proinflammatory cytokines secretion. Adiponectin can suppress Aβ-oligomers induced microglial secretion of TNFα and IL-1β [12]. APN is an adipokine that is predominantly expressed in the liver and adipocytes. A full-length APN (~ 30KDa in human; 24KDa in mouse) consists of a signal sequence, a species-variable region, a collagenous domain, and a globular domain. Different oligomeric forms of APN exist in the plasma. Hydroxylation and glycosylation of the lysine residues within the collagenous domain gives rise to the trimeric APN. The conserved cysteine residue located in the N-terminal variable region (Cys36 in human; Cys39 in mouse) is essential for disulfide bond formation, linking trimeric APN into hexamer or high-molecular-weight APN [13–15]. APN does not express in cerebral neurons and glial cells but the trimers and hexamers are blood–brain barrier (BBB) penetrant [16, 17]. Adiponectin receptors (AdipoR1 and AdipoR2) are abundantly expressed in these cells in the hypothalamus, cortex, and hippocampus [18–20]. We and other groups have recently reported that the levels of low molecular weight APN were reduced in the CNS of AD patients [19, 21]. In addition, aged *APN*^*−/−*^ mice developed AD-like pathology and cognitive impairments. APN deficiency in AD mice exacerbated memory functions and cerebral insulin resistance [22]. Our previous works have demonstrated a cause-to-effect relationship between reduction of cerebral APN levels and AD-like pathology as well as cognitions. We have also revealed that treatment with oral active adiponectin receptor agonist (adipoRon) can reverse the cognitive functions in 5xFAD and *APN*^*−/−*^5xFAD mice. We have shown that adipoRon can cross the BBB, although it has a very short half-life in the blood and the brain [19]. Therefore, the pharmacokinetics of adipoRon in clinical application remains a concern.

Gene therapy holds the efficacious treatment potential for neurodegenerative diseases. Overexpression of neurotrophic or neuroprotective factors by gene delivery using adeno-associated viral (AAV) vectors showed improvement of pathological changes and cognitive functions in transgenic amyloid mice [23, 24]. AAV delivery of APN into the CNS has shown therapeutic benefit in a mouse model of ischemic stroke [25]. However, direct injection of AAV to the brain is invasive and has other negative effects and technical concerns in clinical application. To overexpress APN as a therapeutic strategy for AD, we have generated liver-specific targeting AAV which carries a mutated form APN^C39S^ to overexpress trimeric APN (APN^Tri^). Expression of APN^Tri^ in the liver has shown a dose-dependent increase of AAV administration and increased cerebral APN levels in 5xFAD mice. In this report, we found that APN deficiency in 5xFAD mice exacerbated NLRP3 inflammasome activation and increased IL-1β levels in the brain. Single-dose of AAV-APN^Tri^ can suppress NLRP3 activation and downstream signalling markers, improving spatial learning and memory function in 5xFAD mice. Moreover, overexpression of trimeric APN can reduce amyloid deposition and Aβ-associated dystrophic neurites.

## Results

### Adiponectin deficiency exacerbates microgliosis and induces microglial NLRP3 inflammasome activation in AD mice in an age-dependent manner

**Fig. 1.**
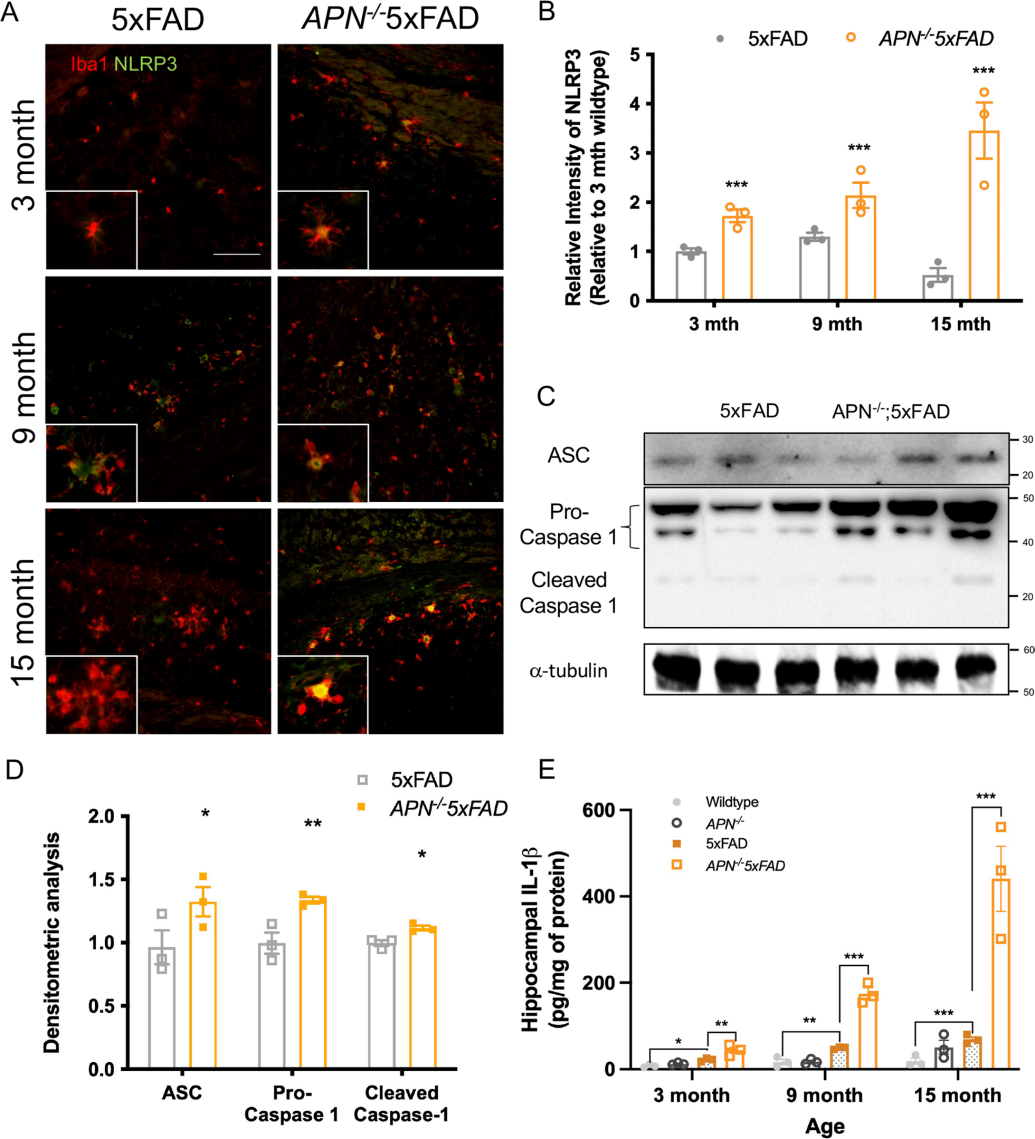
Adiponectin deficiency exacerbates NLRP3-inflammasome activation in 5xFAD mice. **A** Representative images of co-immunofluorescent staining of NLRP3 and Iba1 in the hippocampus of 3, 9, and 15-month-old 5xFAD and *APN*^*−/−*^5xFAD mice. (n = 3 mice for each experimental group) (Magnification: 10 × 20; Magnified: 10 × 40; Scale bar: 50μm). **B** Relative intensity of NLRP3 immunofluorescent staining in the hippocampus. **C** Western blotting assay of ASC, Pro-caspase-1, and cleaved caspase-1 in the hippocampus of 9-month-old 5xFAD and *APN*^*−/−*^5xFAD mice. (n = 3 mice for each experimental group). **D** Densitometric analysis of ASC, Pro-caspase-1, and cleaved caspase-1 levels. **E** ELISA assay of hippocampal IL-1β of 3, 9, and 15-month-old 5xFAD and *APN*^*−/−*^5xFAD mice. (n = 3 mice for each experimental group). Data were presented as the mean ± s.e.m. *p < 0.05; **p < 0.01; ***p < 0.001. Statistical analysis was performed by unpaired t tests.

We and other groups have reported that APN can modulate microglia activity in the CNS. Our team has also shown that activation of APN signalling can increase microglia phagocytosis and reduces IL-1β levels [12]. APN deficiency in 5xFAD mice leads to early Aβ deposition [19]. To examine if APN deficiency leads to the early event of microglial-mediated neuroinflammation, we studied the temporal expression of Iba1, a marker of microglia, in the hippocampus of wild type (WT), *APN*^*−/−*^, 5xFAD, and *APN*^*−/−*^5xFAD mice at the age of 3, 6, 9, 12, 15, 18 months. In the hippocampus, *APN*^*−/−*^5xFAD mice also showed more Iba1 intensity than that of 5xFAD mice from 3 to 9 months. The Iba1 intensity began to drop in *APN*^*−/−*^5xFAD mice from 12 months then decreased at 15 months and 18 months as with *APN*^*−/−*^5xFAD mice (Additional file 1: Fig. S1). This indicates that microgliosis is reduced in aged *APN*^*−/−*^5xFAD compared to 5xFAD mice. NLRP3 inflammasome signalling is crucial to modulate IL-1β secretion in AD brains that become a new therapeutic target to treat AD. Therefore, we studied whether APN deficiency in AD mice led to an increase in IL-1β level which is associated with NLRP3 inflammasome activation. We performed co-immunofluorescent staining of Iba1 and NLRP3 at 3-, 9-, 15-month-old 5xFAD and *APN*^*−/−*^5xFAD mice. The expression of Iba1 in 3-month-old and 9-month-old *APN*^*−/−*^5xFAD was significantly increased in the hippocampus compared to that of the 5xFAD mice (Fig. 1a). Furthermore, microglial NLRP3 expression was also markedly increased in APN-deficient 5xFAD mice from 3 to 15 months (Fig. 1a, b). Though the number of microglia was reduced in 15 month-old *APN*^*−/−*^5xFAD mice, the microglial NLRP3 levels was further increased in APN-deficient 5xFAD compared to 5xFAD mice. This indicates that APN deficiency exacerbates NLRP3 expression in microglia in aging. Western blot analysis has also demonstrated that ASC and caspase-1 levels in the hippocampus were higher in *APN*^*−/−*^5xFAD than in 5xFAD mice (Fig. 1c, d). Lastly, we studied the effect of adiponectin deficiency in IL-1β levels of AD hippocampus. We found that APN deficiency led to increased lL-1β levels in the hippocampus of 5xFAD mice from 3-month to 15-month-old (Fig. 1e). These results indicated that APN deficiency induces microglial NLRP3 inflammasome activation leading to severe neuroinflammatory responses in the AD mouse model.

### Trimeric adiponectin suppresses AβO-induced NLRP3 activation and proinflammatory cytokines secretion from microglia

**Fig. 2:**
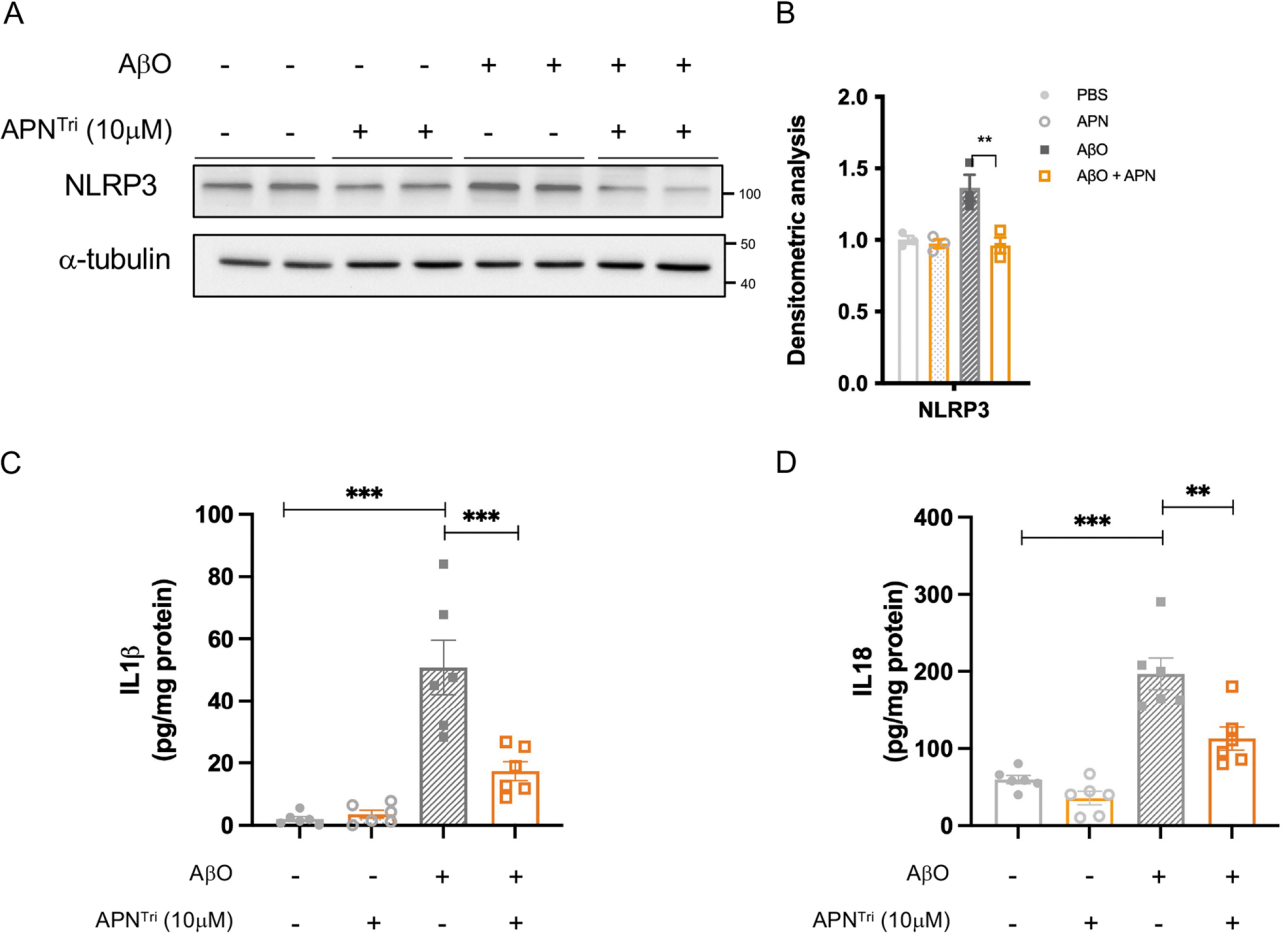
Trimeric adiponectin suppresses Aβ oligomer-induced IL-1β secretion from microglia via NLRP3-inflammsome pathway. **A** Representative western blotting image indicates pre-treatment of mammalian recombinant trimeric APN protein reduced NLRP3 level in Aβ oligomer-induced microglia. (n = 3 cultures for each group with duplicated experiment). **B** Densitometric analysis of NLRP3 and α-tubulin levels. **C** ELISA assay of IL-1β in the microglia BV2 cells. (n = 6 for each group). **D** ELISA assay of IL-18 in the microglia BV2 cells. (n = 6 for each group). Data were presented as the mean ± s.e.m for at least three independent experiments, and each performed in duplicates (*n* = 3). *p < 0.05; **p < 0.01; ***p < 0.001. Statistical analysis was performed by one-way ANOVA followed with Bonferroni’s post hoc comparison tests.

Accumulation of extracellular Aβ stimulates microglia through binding of surface receptors e.g. TLRs promoting NLRP3 inflammasome activation [26, 27]. These receptors promote nuclear translocation of NF-κB to enhance mRNA expression of NLRP3, ASC, caspase-1, and pro-IL-1β [28]. In our previous report, we have shown that APN can suppress AβO-induced NF-κB nuclear translocation through the activation of AdipoR1-AMPK in BV2 microglia [12]. To examine if APN can reduce NLRP3, ASC, and pro-caspase-1 levels, the BV2 microglia was pre-treated with or without recombinant trimeric APN for 3 h followed by AβO incubation. We found that the NLRP3 levels were significantly increased in BV2 microglia after incubating with AβO for 48 h. Western blot analysis revealed that AβO significantly increased the levels of NLRP3 in BV2 microglia. Trimeric APN treatment could suppress the level of NLRP3 suggesting APN can inhibit NLRP3-inflammasome activation under Aβ stimulation (Fig. 2a, b). Caspase-1 cleaves premature IL-1β and IL-18 into mature IL-1β and IL-18 in microglia prior to secretion. To examine if trimeric APN can suppress NLRP3-inflammasome pathway and mature IL-1β and IL18 in BV2 microglia, we then examined the level of IL-1β and IL-18 in the cell culture medium by ELISA assay. We found that AβO significantly increased the levels of IL-1β and IL-18 in the microglia BV2 whereas trimeric APN treatment reduced the levels of IL-1β and IL-18 (Fig. 2c, d). This data fills the gap of how APN-AMPK-NF-κB cascade modulate IL-1β levels in microglia. These results suggested that mammalian trimeric APN protein could suppress AβO-induced microglial NLRP3-inflammasome activation and IL-1β and IL-18 level in microglial cells.

### Systemic administration of AAV shows a liver-specific expression of trimeric APN and increases in cerebral APN levels in AD mice

**Fig. 3:**
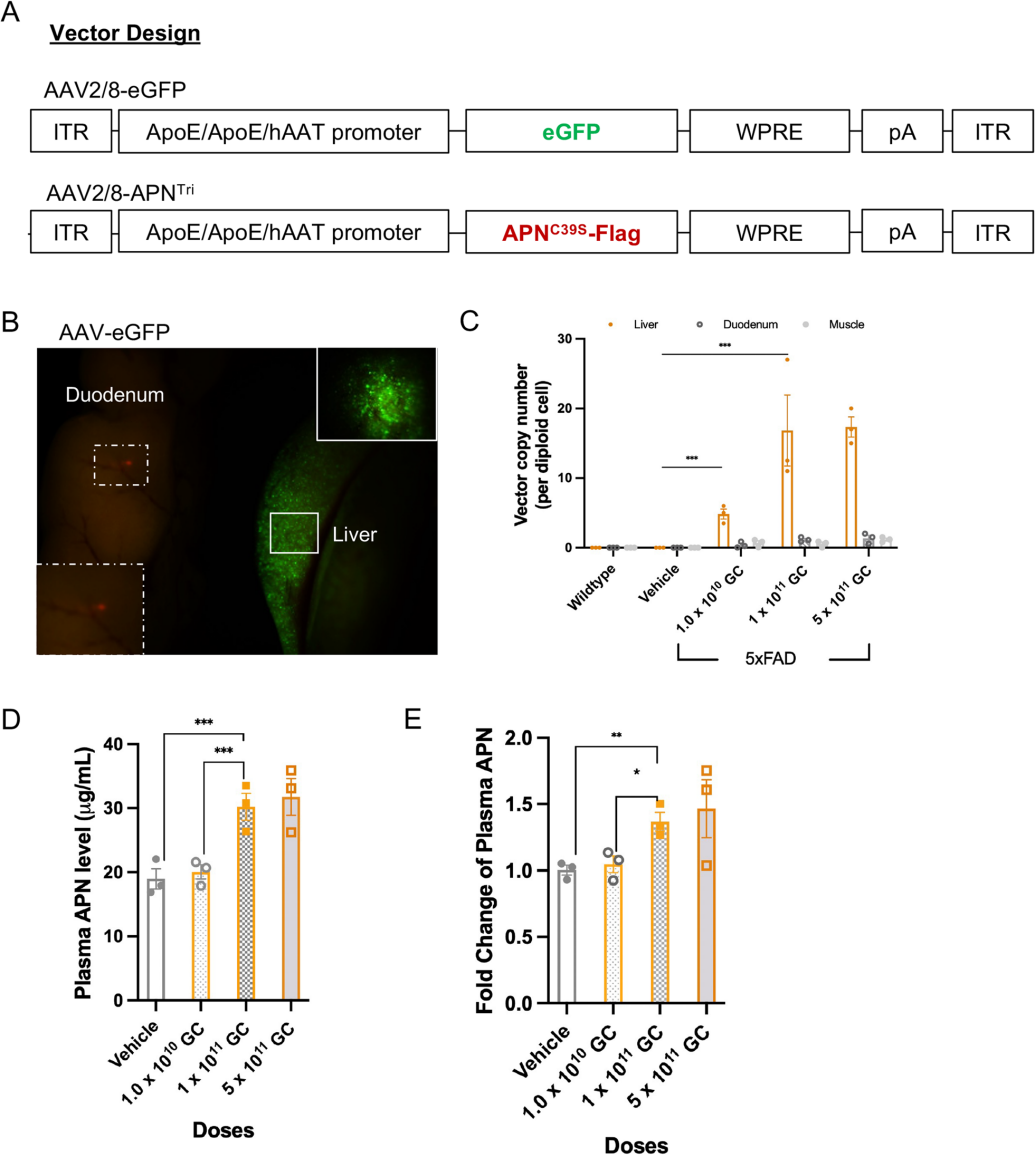
AAV2/8-mediated gene delivery of *APN*^*C39S*^ transgene to 5xFAD exhibits dose-dependent transduction efficiency, liver-specific expression of transgene and increase of circulatory adiponectin. **A** AAV plasmids design of control GFP and APN^C39S^. **B** Fluorescence of GFP in the liver and duodenum of AAV2/8-GFP-treated 5xFAD 1 month after injection. (n = 3 mice). **C** Vector copy number of APN transgene in the liver, duodenum and muscle of AAV2/8-APN^Tri^-treated 5xFAD at different doses 1 month after injection. (n = 3 mice for each experimental group). **D** ELISA analysis of plasma APN in AAV2/8-APN^Tri^-treated 5xFAD at different doses 1 month after injection. (n = 6 mice for each experimental group). **E** Fold change analysis of plasma APN in AAV2/8-APN^Tri^-treated 5xFAD at different doses 1 month after injection (normalized by wildtype plasma APN). Data were presented as the mean ± s.e.m. *p < 0.05; **p < 0.01; ***p < 0.001. Statistical analysis was performed by one-way ANOVA followed with Bonferroni’s post hoc comparison tests.

It has been reported that APN is not expressed in the brain and only low-molecular-weight APN can penetrate the BBB [16]. Our previous studies demonstrated that low-molecular-weight APN (trimeric and hexameric) levels are reduced in AD brains [19]. This indicates that reduction of low-molecular-weight APN is associated with AD. To increase the level of low-molecular-weight APN in the brain of AD mice, we produced liver-specific AAV serotype 2/8 encoding trimeric APN (AAV2/8-APN^Tri^) or control green-fluorescent GFP protein (AAV2/8-GFP). The AAV2/8 vector carries mutated adiponectin C39S cDNA which prevents the overexpressed APN to form a higher-order complex beyond the trimeric complex. A flag sequence was linked at the C-terminal of the APN^C39S^ cDNA to distinguish it from the endogenous mouse APN in the latter analysis. This AAV vector expresses APN under the control of APOE/APOE/hAAT promoter to achieve liver-specific expression (Fig. 3a). We injected the AAV2/8-GFP particles into AD mice via tail vein. We found that AAV provide robust transgene expression in the liver but not in other peripheral organs 1 month after injection (Fig. 3b). Next, we determined the in vivo efficacy of AAV2/8-APN^Tri^ by systemic administration in 5xFAD mice. The 4-month old 5xFAD mice were treated with a single intravenous injection with either the control AAV2/8-GFP or AAV2/8-APN^Tri^ at doses of 1.0 × 10^10^ GC, or 1 × 10^11^ GC, or 5.0 × 10^11^ GC. To evaluate the transduction efficiency of AAV2/8-APN^Tri^ in the liver, we measured AAV vector genome copy numbers by a real-time PCR test in different periphery tissues collected from the euthanized mice 1 month after AAV treatment. We observed a dose-dependent increase in the vector copy in the liver but not in the muscle or duodenum. The vector genome copies at doses of 1 × 10^11^ GC (16.8 ± 5.1 vg/dg) and 5.0 × 10^11^ GC (17.3 ± 1.5 vg/dg) were significantly higher than that at the dose of 1 × 10^10^ GC (4.8 ± 0.7 vg/dg) (Fig. 3c), that also translated into dose-dependent increase in the plasma APN levels (Fig. 3d). Plasma was collected from each mouse before injection and 1 month after AAV administration. The fold change of plasma APN was significantly higher at the dose of 1 × 10^11^ GC (1.04 ± 0.06) than that at the dose of 1 × 10^10^ GC (1.37 ± 0.07) (Fig. 3e). There was no appreciable increase of the vector genome copies and plasma APN between the doses of 1 × 10^11^ GC and 0.5 × 10^12^ GC.

It has been shown that the recombinant AAV2/8 liver specific virus would not induce vector-mediated hepatotoxicity after single dose injection in mice. No significant changes of serum alanine amino transferase level and tumour observed in the mouse livers [29]. In our experiment, recombinant AAV2/8 did not cause significant changes of the body weight, weight changes, and liver weight 20 weeks after administration (Additional file 1: Table S1). In the previous analysis, no immune cell infiltration was induced in the liver after AAV2/8 administration [29]. Similarly, we found no infiltration of mononuclear cells from the blood capillaries into the liver after AAV gene delivery indicating this method is safe (Additional file 1: Fig. S2). All the mice survived until the end-point of the experiment. These results indicated that AAV2/8-APN^Tri^ has no overt toxicity in the preclinical model and holds treatment potential that can specifically increase liver APN and plasma APN 1 month after AAV injection.

**Fig. 4:**
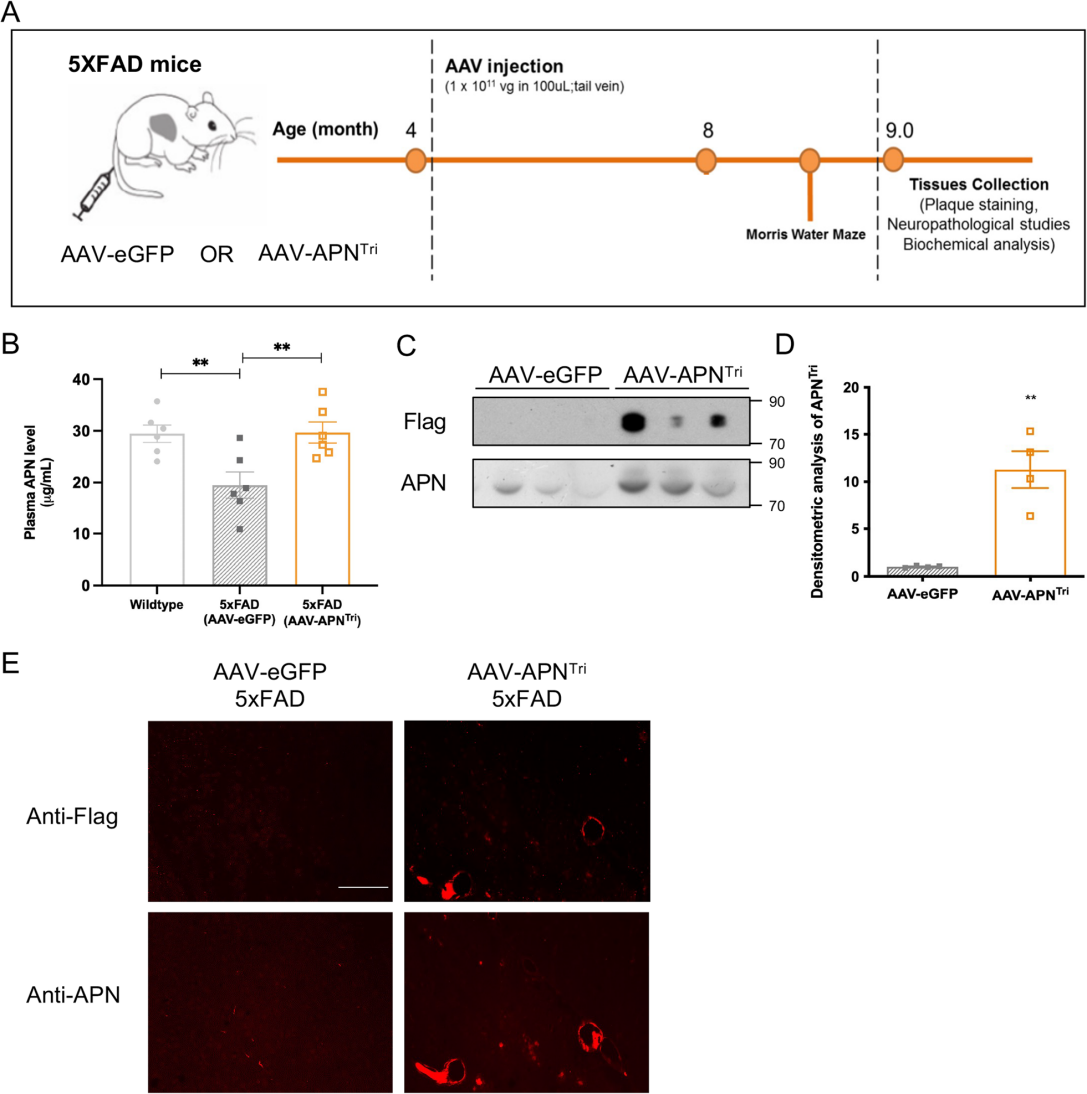
Single dose of AAV2/8-APN^Tri^ increases trimeric adiponectin in circulation and cerebral adiponectin levels in 5xFAD mice 4 months after injection. **A** Preclinical study scheme of single dose AAV injection at 4-month-old 5xFAD. **B** ELISA analysis of plasma APN in wildtype, AAV2/8-GFP-treated, and AAV2/8-APN^Tri^-treated 5xFAD 4 month after injection. (n = 6 mice for each experimental group). **C** Non-denaturing SDS PAGE analysis of plasma APN by anti-flag and anti-APN antibodies indicated the levels of trimeric APN in the circulation 4 months after injecting AAV2/8-GFP and AAV2/8-APN^Tri^. (n = 4 mice for each experimental group). **D** Densitometric analysis of APN^Tri^ in AAV2/8-GFP-treated and AAV2/8-APN^Tri^-treated 5xFAD. **E** Immunofluorescent staining of APN using anti-flag and anti-APN antibodies in the cerebral cortex of AAV2/8-GFP-treated, and AAV2/8-APN^Tri^-treated 5xFAD 4 month after injection. (n = 4 mice for each experimental group) (Magnification: 10 × 20; Scale bar: 50μm). Data were presented as the mean ± s.e.m.; *p < 0.05; **p < 0.01; ***p < 0.001. Statistical analysis was performed by one-way ANOVA followed with Bonferroni’s post hoc comparison tests and unpaired t test.

To study whether liver-specific overexpression of trimeric APN results in an increased level of cerebral APN for chronic treatment of AD, the single dose at 1 × 10^11^ GC was chosen for intravenous injection into 4-month old 5xFAD mice (Fig. 4a). The treatment strategy relies on the circulation to transport trimeric APN from the liver to the CNS. AAV2/8-GFP treated 5xFAD mice had lower plasma APN levels than WT mice (19.50 μg/mL ± 2.546 vs 29.45μg/mL ± 1.686). In contrast, AAV2/8-APN^Tri^ treatment increased the level of plasma APN in 5xFAD mice that was higher than the AAV2/8-eGFP-treated 5xFAD mice (29.69 μg/mL ± 2.027 vs 19.50 μg/mL ± 2.546) (Fig. 4b). To confirm AAV2/8-APN^Tri^ injection increases circulatory trimeric APN in 5xFAD mice, we performed non-denaturing SDS PAGE followed with western blot analysis of anti-APN and anti-flag. We found that AAV2/8-APN^Tri^ injection significantly increased the level of trimeric APN in the circulation of 5xFAD mice (Fig. 4c, d). The trimeric APN detected by the anti-flag antibody, which distinguished APN from the endogenous protein, further confirmed the expression was specific from the AAV2/8-APN^Tri^ vector. Finally, we examined if the overexpressed APN could reach the brain. Immunofluorescent staining of APN and flag on brain sections indicated that APN level was increased in the brain after AAV2/8-APN^Tri^ injection (Fig. 4e). The anti-flag immunofluorescent staining further indicated that the increase of APN in the brain was trimeric APN. These results demonstrate liver-specific delivery of AAV2/8 overexpresses trimeric APN in the brain through the circulation.

### Trimeric APN improves cognitive functions and ameliorates neurodegeneration

**Fig. 5:**
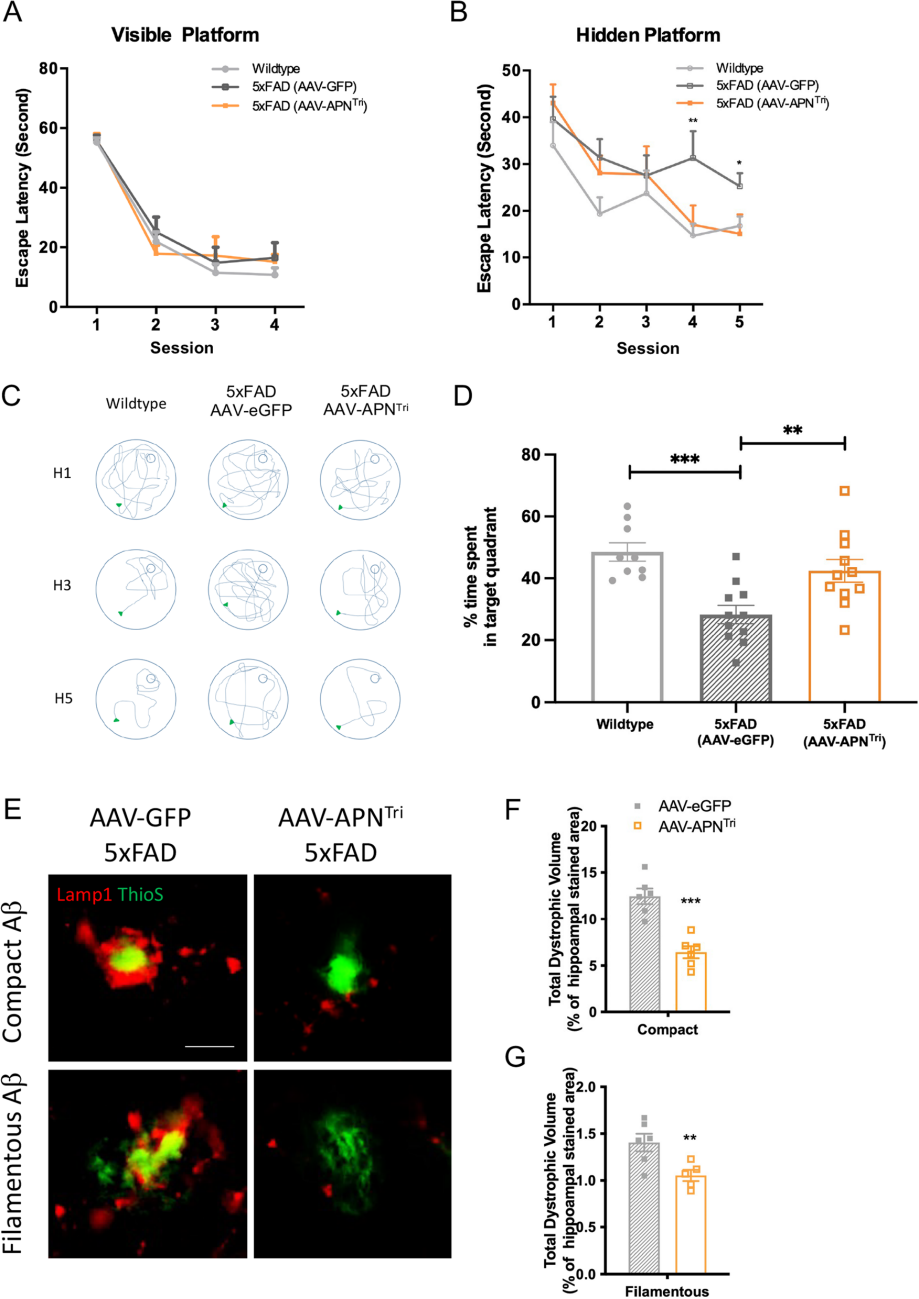
AAV2/8-APN^Tri^ treatment improve memory functions and reduces dystrophic neurites in the 5xFAD mice. **A** The escape latency of different treatment group in the visible platform of Morris water maze (MWM) test. (n = 9 mice for wildtype and n = 11 for both AAV-treated groups). **B** The escape latency in 5-day sessions performed by different treatment group in MWM test. (n = 9 mice for wildtype and n = 11 for both AAV-treated groups). **C** Representative tracks of different treatment groups in the hidden sessions (H1, H3 & H5). **D** Probe test indicates the percentage of time spent in the target quadrant (platform location) by different mouse treatment groups. (n = 9 mice for wildtype and n = 11 for both AAV-treated groups). **E** Representative images of co-staining of Aβ and Lamp1 in the cortex and hippocampus of AAV2/8-GFP-treated (n = 6), and AAV2/8-APN^Tri^-treated 5xFAD (n = 6) (Total 780 plaques were analyzed; Magnification: 10 × 40; Scale bar: 10μm). **F**, **G** Quantification of total dystrophic volume represented by Lamp1 staining surrounding the compact Aβ and filamentous Aβ. Data were presented as the mean ± s.e.m. *p < 0.05; **p < 0.01; ***p < 0.001. Statistical analysis of MWM tests (A, B) was analyzed with two-way ANOVA followed by Bonferroni’s post hoc test. Statistical analysis in (**D**) was performed by one-way ANOVA followed with Bonferroni’s post hoc comparison tests and in (**F**, **G**) were performed by unpaired t test.

We first tested whether overexpression of periphery trimeric APN by liver-specific AAV2/8 delivery can improve cognitive performance. Mice were subjected to the Morris water maze test to examine the changes in spatial learning and memory functions. There was no significant difference of escape latency across different experimental group in the visible platform sessions (Fig. 5a). In the hidden platform test, AAV2/8-GFP injected 5xFAD mice showed impaired learning and memory acquisition compared to the wildtype control (Fig. 5b). Control 5xFAD had longer escape latency compared to the wildtype mice in the last session of the hidden test (25.27 s ± 2.802 vs 16.83 s ± 2.034). Importantly, overexpression of trimeric APN by AAV reduced the escape latency of 5xFAD mice indicating AAV2/8-APN^Tri^ treated mice had improved spatial learning ability than AAV2/8-GFP treated 5xFAD mice (15.11 s ± 4.125 vs 25.27 s ± 2.802). The representative track of mice in the last session of the hidden platform test also indicated that the AAV2/8-APN^Tri^ treated 5xFAD mice had better performance in locating the platform (Fig. 5c). Probe test has been used to examine the memory retention of mice. AAV2/8-eGFP-treated mice have significant reduced percentage of time spent in the target quadrant compared to the wildtype mice (28.70% ± 3.608 vs 48.36% ± 2.959). AAV2/8-APN^Tri^ treated 5xFAD mice spent a higher percentage of time in the target quadrant compared with the control 5xFAD mice (43.63% ± 4.351 vs 28.70% ± 3.608). This indicated that the AAV2/8-APN^Tri^ treated 5xFAD mice had better memory retention than the control 5xFAD mice (Fig. 5d). Average swim speed and distance were unchanged across different groups in the probe test indicating no difference in motor function (Additional file 1: Fig. S3a, b). This data supports a therapeutic effect for trimeric APN in learning and memory.

Dystrophic neuron is a common AD pathology that leads to neuronal dysfunction and memory impairment. Lamp1 is a marker of neuronal dystrophy that robustly demarcate the swollen dystrophic axons around Aβ deposits by confocal microscopy [30–32] and is a common pathology in AD brains [33]. To determine if neuronal dystrophy has been improved after AAV2/8-APN^Tri^ treatment, the brain sections were co-stained with thioflavin S and Lamp1. We then defined the filamentous non-compact plaque and compact plaque (circular upto 6μm in diameter) as previously described [34]. We found that the volumes of filamentous Aβ- and compact Aβ-associated Lamp1 were reduced in AAV2/8-APN^Tri^ treated 5xFAD mice compared with the control 5xFAD mice (Fig. 5e–g). The levels of synaptic protein such as spinophilin have also significantly increased in 5xFAD mice after AAV2/8-APN^Tri^ treatment. However, the level of synaptophysin was insignificantly increased (Additional file 1: Fig. S3c, d). The results implicated that the number of Aβ-associated dystrophic neurons was reduced after AAV2/8-APN^Tri^ treatment. Taken altogether, AAV2/8-APN^Tri^ treatment attenuates neuronal injury and improves cognitive functions of 5xFAD mice.

### Trimeric APN reduces Aβ deposition and microglial activation

**Fig. 6:**
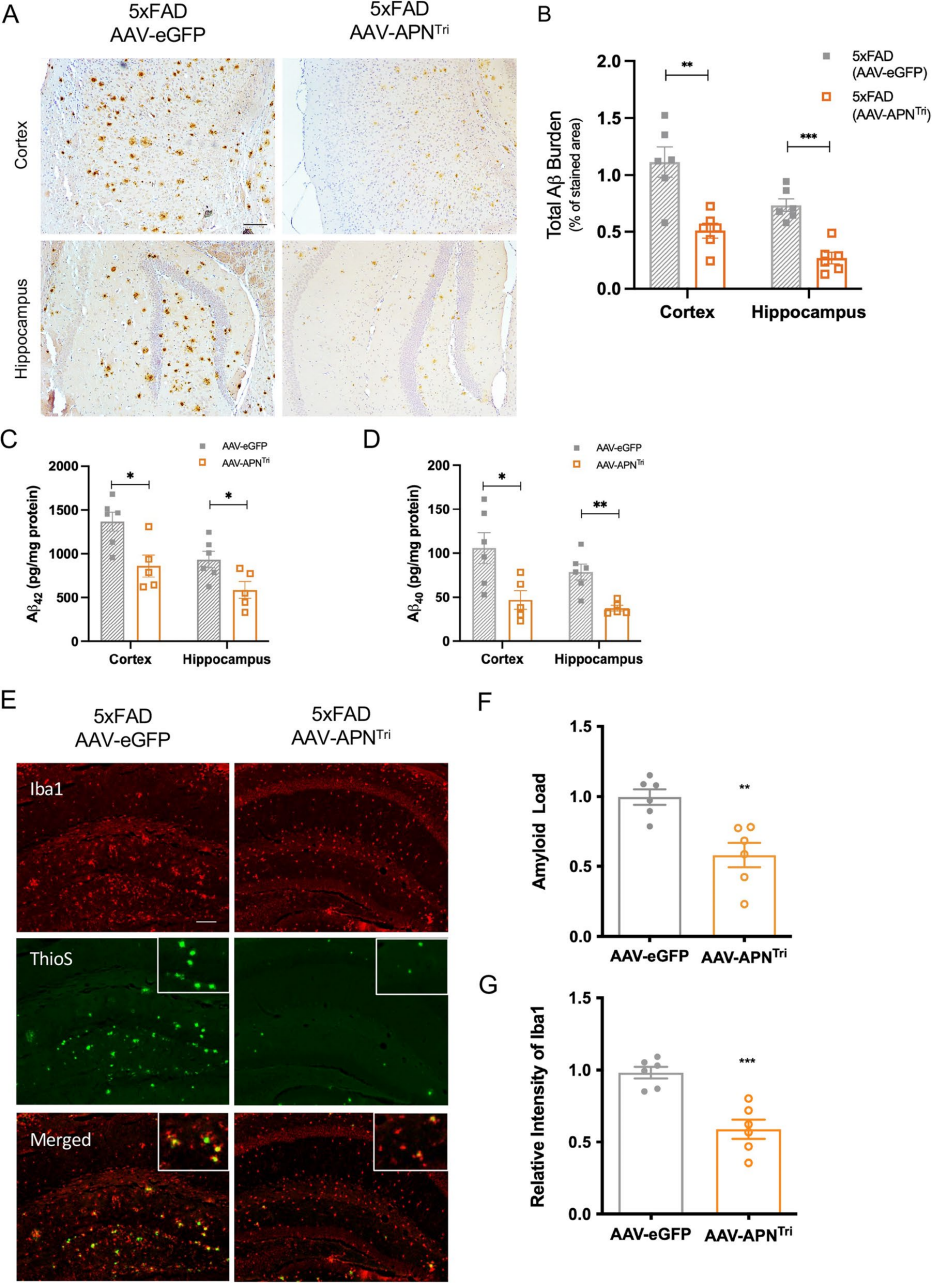
AAV2/8-APN^Tri^ treatment reduces amyloid pathology and microglia activation in the 5xFAD mice. **A** Immunohistochemistry analysis of Aβ (4G8; brown) in the hippocampus and cerebral cortex of AAV2/8-GFP-treated, and AAV2/8-APN^Tri^-treated 5xFAD. (n = 6 mice for each experimental group) (Magnification: 10 × 10; Scale bar: 50μm). **B** Quantitative analysis of the Aβ loading and number of Aβ deposits in the hippocampus and cortex. **C**, **D** ELISA analysis of Aβ_42_ and Aβ_40_ levels in the hippocampus and the frontal cortex of AAV2/8-GFP-treated, and AAV2/8-APN^Tri^-treated 5xFAD. (n = 5-6 mice for each experimental group). **E** Iba1 (red) and thioflavin S (green) double-immunofluorescent staining in the hippocampus of AAV2/8-GFP-treated, and AAV2/8-APN^Tri^-treated 5xFAD. (n = 6 mice for each experimental group) (Magnification: 10 × 10; Scale bar: 50μm). **F** Quantitative analysis of the Aβ loading in the hippocampus of AAV2/8-GFP-treated, and AAV2/8-APN^Tri^-treated 5xFAD. **G** Relative intensity of Iba1 in the hippocampus of AAV2/8-GFP-treated, and AAV2/8-APN^Tri^-treated 5xFAD. Data were presented as the mean ± s.e.m. n.s. not significant; *p < 0.05; **p < 0.01; ***p < 0.001. Statistical analysis was performed by unpaired t tests.

Aβ is the pathological hallmark of AD that causes microglia activation and neuronal injury. Since overexpression of trimeric APN in the liver can increase APN levels in mouse brains, we then studied if overexpression of trimeric APN in the liver can reduce Aβ deposition in AD mouse brain 4 months after AAV injection. By immunohistochemistry of Aβ, amyloid load (percentage of stained area) in the hippocampus and cerebral cortex of 5xFAD mice at 9 months of age were 0.7361% ± 0.057 and 1.115% ± 0.133 respectively, whereas the amyloid load in the hippocampus and cerebral cortex of AAV-APN^Tri^-treated mice were 0.2694% ± 0.051 and 0.5102% ± 0.066. The AAV-APN^Tri^ treatment had reduced the amyloid load by 63.4% in the hippocampus and 54% in the cortex (Fig. 6a, b). The Aβ_40_ and Aβ_42_ peptides assessed by enzyme-linked immunosorbent assay decreased by 50% ± 3 and 57% ± 4, respectively, in pooled cerebral cortex and hippocampus samples (Fig. 6c, d).

To examine if AAV-APN^Tri^ treatment reduced Aβ-associated microglial activation in the brain of 5xFAD, we performed double immunofluorescence staining of Aβ and microglia by thioflavinS and Iba1. We found that the overall levels of thioflavin-stained fibrillar Aβ were decreased in both the cortex and the hippocampus (Fig. 6e and Additional file 1: Fig S4a). The number of Aβ plaque and size of plaques was also significantly reduced in the hippocampus after AAV-APN^Tri^ treatment (Additional file 1: Fig. S4a, b). The relative intensity of Iba1 was reduced indicating that AAV-APN^Tri^ suppressed microgliosis of 5xFAD mice. Astrogliosis is also one of the common AD pathologies. AAV-APN treatment can reduce Aβ-associated astrogliosis in both the cortex and hippocampus in the 5xFAD mice than the control 5xFAD mice (Additional file 1: Fig. S4c, d). These results demonstrate that overexpression of trimeric APN can reduce AD pathology in the AD mouse model.

**Fig. 7:**
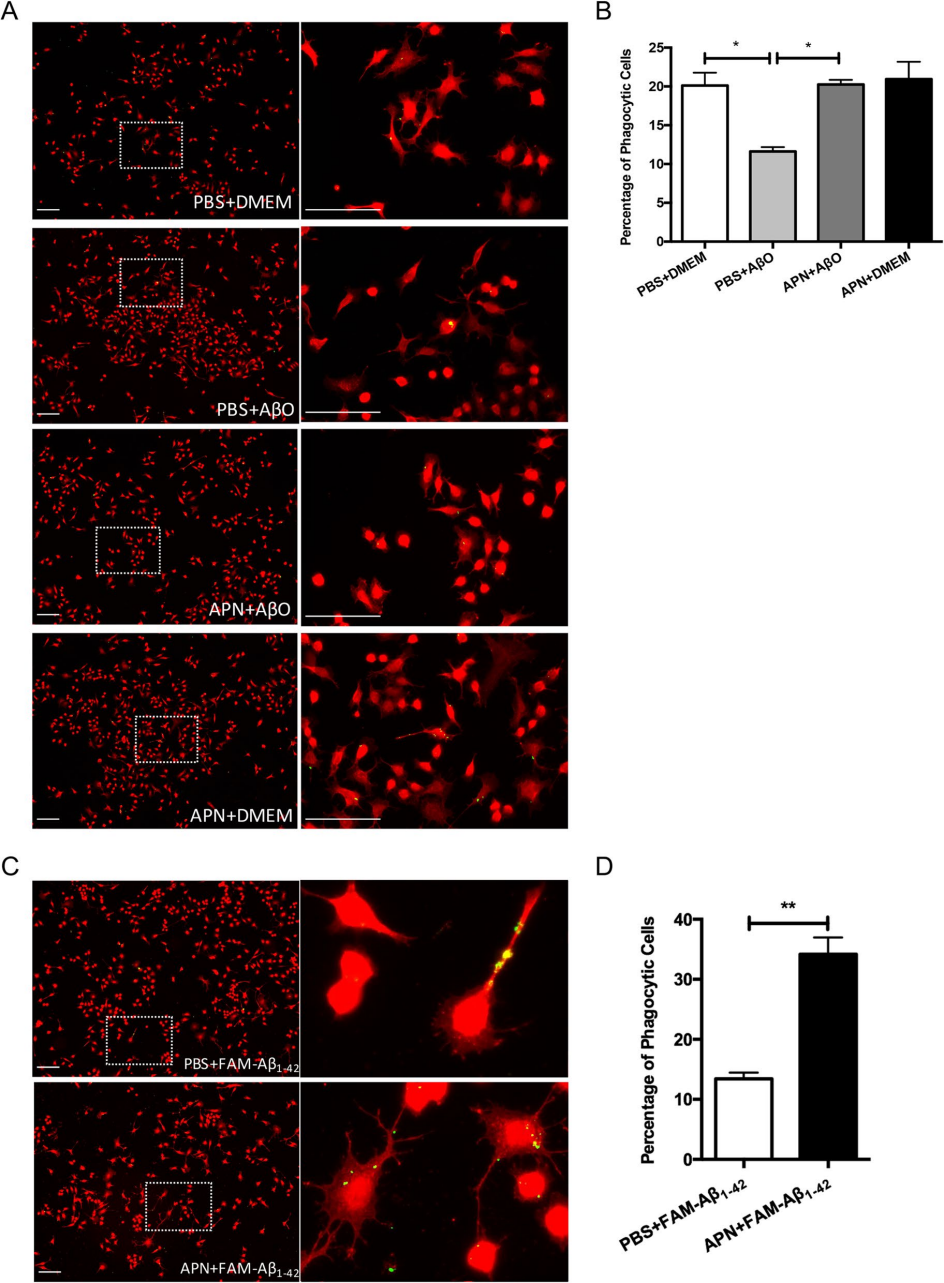
Recombinant APN increases microglial phagocytic activities induced by Aβ peptides and oligomers in vitro. **A** Representative images of FAM-Aβ_1-42_ phagocytosis in BV2 microglia cells. BV2 cells were pre-incubated with APN for 2 h followed by incubation with FAM-Aβ_1-42_ (green) for 24 h before staining Iba1 (red). Scale bar = 100 μm. **B** The bar graphs showed percentage of phagocytic cells Data were presented as the mean ± SEM for at least three independent experiments (n = 3). **C** Representative images of latex bead phagocytosis in BV2 microglia cells. BV2 cells were pre-incubated with APN for 2 h followed by incubation with AβO for 24 h and then were loaded with fluorescent beads (green) for 1 h at 37 °C before staining Iba1 (red). Scale bar = 100 μm. **D** The bar graphs show percentage of phagocytic cells Data were presented as the mean ± SEM for at least three independent experiments (n = 3). One-way ANOVA with Tukey’s multiple comparison test revealed difference between groups. *p < 0.05

We have previously shown that adipoRon increased microglia phagocytic activity in vivo. To provide evidence that trimeric APN directly influences phagocytic ability of microglia in Aβ removal, the BV2 microglia were pretreated with mammalian trimeric adiponectin for 2 h followed by the treatment of AβO for 24 h, then proceed to phagocytosis assay using fluorescent latex beads. AβO decreased the number of phagocytosed beads in microglia, compared with vehicle group. Trimeric APN significantly rescued the decrease of phagocytosis ability of microglia under AβO exposure. (Fig. 7a, b) To further confirm whether APN regulated microglial phagocytosis on Aβ, we then access the effect of trimeric APN on microglial Aβ_1-42_ phagocytosis. BV2 microglia were treated with trimeric APN for 2 h and subsequently exposed to aggregated FAM-labelled Aβ_1-42_ for 24 h. We found that trimeric APN significantly increased FAM-labelled Aβ_1-42_ phagocytosis in BV2 microglia compared to PBS-pretreated microglia (Fig. 7c, d).

### Trimeric APN reduces microglial NLRP3-inflammasome activation and cerebral IL-1β levels in 5xFAD

**Fig. 8:**
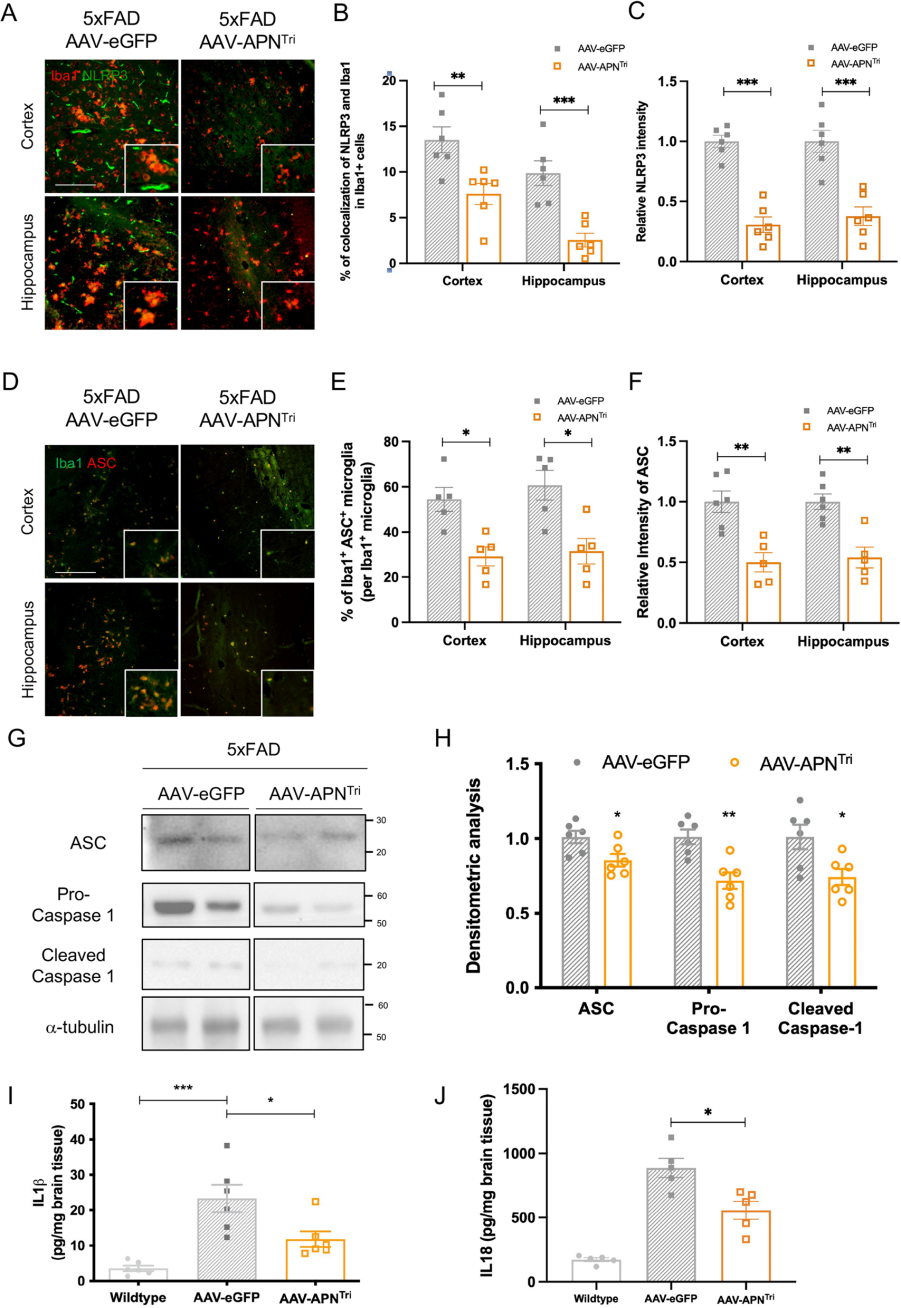
AAV2/8-APN^Tri^ treatment reduces NLRP3-inflammasome activation in the 5xFAD mice. **A** Iba1 (red) and NLRP3 (green) double-immunofluorescent staining in the hippocampus and cortex of AAV2/8-GFP-treated, and AAV2/8-APN^Tri^-treated 5xFAD. (n = 6 mice for each experimental group) (Magnification: 10 × 20; Scale bar: 50μm). **B** Relative intensity of microglial NLRP3 in the hippocampus and cortex of AAV2/8-GFP-treated, and AAV2/8-APN^Tri^-treated 5xFAD. **(C)** Relative intensity of NLRP3 in the hippocampus and cortex of AAV2/8-GFP-treated, and AAV2/8-APN^Tri^-treated 5xFAD. **(D)** Iba1 (green) and ASC (red) double-immunofluorescent staining in the hippocampus and cortex of AAV2/8-GFP-treated, and AAV2/8-APN^Tri^-treated 5xFAD. (n = 6 mice for each experimental group) (Magnification: 10 × 20; Scale bar: 50μm). **E** Percentage of Iba1 + ASC + cells in the hippocampus and cortex of AAV2/8-GFP-treated, and AAV2/8-APN^Tri^-treated 5xFAD. **F** Relative intensity of microglial ASC in the hippocampus and cortex of AAV2/8-GFP-treated, and AAV2/8-APN^Tri^-treated 5xFAD. **(G)** Western blotting assay of ASC, Pro-caspase-1, and cleaved caspase-1 in the hippocampus of AAV2/8-GFP-treated and AAV2/8-APN^Tri^-treated 5xFAD. (n = 5 mice for each experimental group). **H** Densitometric analysis of ASC, pro-caspase-1, and cleaved caspase 1. **(I)** ELISA assay of hippocampal IL-1β of AAV2/8-GFP-treated and AAV2/8-APN^Tri^-treated 5xFAD. (n = 6 mice for each experimental group). **(J)** ELISA assay of hippocampal IL-18 of AAV2/8-GFP-treated and AAV2/8-APN^Tri^-treated 5xFAD. (n = 5 mice for each experimental group). Data were presented as the mean ± s.e.m. n.s. not significant; *p < 0.05; **p < 0.01; ***p < 0.001. Statistical analyses in (B, C, E, F, G, and H) were performed by unpaired t tests and (I, J) were performed by one-way ANOVA followed with Bonferroni’s post hoc comparison tests.

APN deficiency in 5xFAD mice exacerbated microglial activation, NLRP3-inflammasome activation, and IL-1β secretion. We next examined whether AAV-APN^Tri^ treatment reduced neuroinflammation-related protein expression in the brains of AD mice. We found that NLRP3 was highly expressed in both the microglia and cerebral blood vessels of AAV-GFP-treated 5xFAD mice (Fig. 8a). Notably, the microglial NLRP3 expression was also significantly reduced after AAV-APN^Tri^ treatment (Fig. 8a, b). AAV-APN^Tri^ treatment also reduced the overall NLRP3 levels in the cerebral cortex and hippocampus of 5xFAD mice (Fig. 8c). We reasoned that the NLRP3-ASC-inflammasome was therefore suppressed by trimeric APN. We examined the microglial ASC levels in the mice by co-immunofluorescent staining of Iba1 and ASC. We found that AAV-APN^Tri^ treatment reduced the percentage of Iba^+^ASC^+^ microglia (Fig. 8d, e). The overall ASC expression was also reduced after AAV-APN^Tri^ treatment (Fig. 8f). Western blot analysis also indicated that the ASC, caspase-1, and cleaved caspase-1 were reduced after AAV-APN^Tri^ treatment (Fig. 8g, h). AAV-APN^Tri^ treatment also reduced the cerebral IL-1β and IL-18 by 52% and 30% respectively (Fig. 8i, j). These results suggested that liver-specific expression of trimeric APN can inhibit microglial-NLRP3-inflammasome activation and neuroinflammatory Iβ cytokine level in AD.

## Discussion

**Fig. 9:**
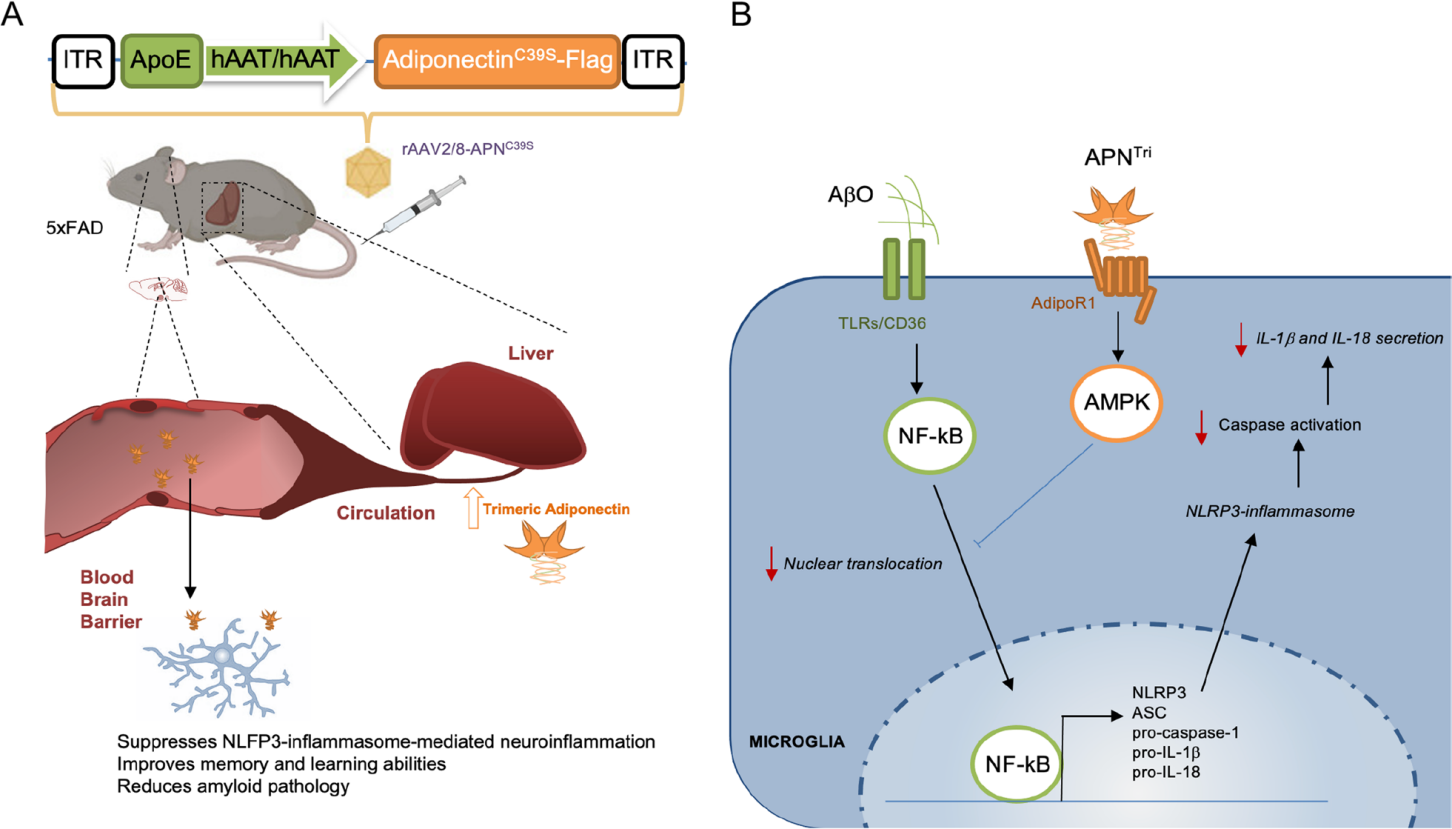
Schematic summary of this study. **A** Strategy of liver-specific adiponectin gene delivery using recombinant AAV as a treatment for Alzheimer’s disease. Intravenous injection of AAV2/8-APN^Tri^ which carries APN mutant (C39S) under the ApoE enhancer and hAAT promoter overexpresses trimeric adiponectin in the liver. Increasing circulatory trimeric APN which crosses the blood–brain barrier to inhibits microglia-mediated neuroinflammation. **B** Molecular mechanism of the model. AβO stimulates nuclear translocation of NF-κB through binding of toll-like receptors (TLRs) or CD36. Trimeric adiponectin binds to adiponectin receptor 1 (AdipoR1) and activates AMPK to suppress NF-κB nuclear translocation. This decreases the mRNA transcriptions and the levels of NLRP3, ASC, pro-caspase 1, IL-1β, and IL-18

In this study, we sought to test the efficacy of AAV-mediated *APN* expression in AD-related pathology including amyloid deposition, microglial-mediated neuroinflammation, and dystrophic neurites observed in APP and PSEN1 transgenic mice with 5 AD-linked mutations. We have shown that overexpression of trimeric APN in the liver can increase the circulatory trimeric APN and cerebral APN. We reason that the periphery delivery of APN^C39S^ mutant gene, in particular the liver, can be an alternative route to increase trimeric APN level in the brain for the treatment of AD (Fig. 9a).

Gene delivery by AAV is a safe method but is challenging in treating CNS disorders. The approach of AAV gene therapy of neurological diseases usually requires intracerebral injection of AAV particles. While direct AAV delivery into the brain parenchyma using stereotaxic surgery is safe and efficient in many preclinical models of neurological disease, it is highly invasive to translate into human. The vector spread is also limited despite axonal transport facilitating anatomical AAV distribution [35]. Intraventricular and intracisternal delivery of AAV improves widespread expression but they are invasive procedures, which requires the considerable surgical skills and knowledge of brain infrastructure [36]. Systemic injection of AAV may also trigger human neutralizing anti‐AAV antibodies that can significantly reduce the transduction rate in the CNS [37, 38]. The requirement of BBB penetration also limited the use of AAV tropism. Recently, the liver becomes an attractive organ for the development of gene-based therapy for several reasons including: (1) it is one of the body’s major biosynthetic organs; (2) studies in small, large-animal models, and humans have demonstrated that it is possible to target hepatocytes with adequate efficiency using intravenously administered AAV vectors; (3) transgene expression last over years after gene transfer to the liver in large animals and humans. (4) expression of a transgene in hepatocytes induces antigen-specific tolerance mediated by regulatory T cells (Tregs). Clinical studies with AAV gene transfer of *factor IX* (*FIX*), the blood coagulation protein, were successfully expressed under the control of a liver-specific promoter in hemophilia patients [39, 40]. Another study has demonstrated single dose of AAV-human factor VIII can provide a sustainable clinical benefit to patients with hemophilia A [41]. Our group has demonstrated AAV2/8 serotype is highly liver selective and the use of ApoE/ApoE/hAAT liver-specific promoter leads to high expression of the transgene APN^C39S^. APN is predominantly expressed in adipocytes and hepatocytes. The overexpression of trimeric APN in the transfected liver would not cause obvious mononuclear cell infiltration at the end-point of our study, indicating this approach did not induce immunogenicity, at least in the animal model. In clinical trial, administration of glucocorticoids after liver-specific vector treatment can help to prevent and treat AAV capsid immune response [42]. Therefore, the application of liver-specific AAV based gene therapy is well tolerated.

Microglia and astrocyte activation in the AD brain release proinflammatory cytokines (e.g. TNFα, IL-6, IL-18, and IL-1β) that promote neurodegeneration. Loss-of-function studies have revealed the essential roles of APN and NLRP3 in microglial-mediated neuroinflammation and cognitive functions. APN deficiency and NLRP3 inflammasome activation are known to induce IL-1β secretion in pathological conditions. In our study, depletion of APN in transgenic amyloid mice significantly increases NLRP3, ASC, and IL-1β from 3 to 15 months, whereas NLRP3 expression increased from 3 to 9 months but reduced from 9 to 15 months in 5xFAD mice even though IL-1β level was increased. Although the reason for such difference is unclear, it is conceivable that the longer period of amyloid deposition in the case of the 9- to the 15-month group may cause irreversible alterations in the induction of pro-inflammatory innate immune pathways which are either NLRP3-independent or non-responsive to amyloid. It has been shown that NLRP1, which is primarily found in both neurons and microglia, is also significantly increased in AD brains [43]. NLRC4 is another inflammasome that has been recently found upregulated in the AD brains [7]. All these suggest that NLRP3-inflammasome is critical but may not be the only inflammasome to process IL-1β in AD. NLRP3 selective inhibitors (e.g. MCC950, OLT1177) that have shown effective for reversing AD pathology in amyloid mouse models [8, 11], might not be enough to suppress IL-1β levels and the detrimental effects of IL-1β in human AD. NF-κB translocation into the nucleus increases NLRP3 and IL-1β expression that is associated with neuroinflammation in the pathophysiology of AD [8, 44–46]. NLRP3 expression induced by NF-κB is the first priming signal before NLRP3 inflammasome activation [47]. In our previous study, we have demonstrated that APN can inhibit the AβO-induced nuclear translocation of NF-kB and reduce the expression of IL-1β in microglia [12]. In the current study, we have also shown that trimeric APN can reduce the expression of NLRP3 in microglia under AβO induction. Collectively, activation of APN signaling can suppress Aβ induced NF-kB-NLRP3-IL-1β pathway in microglia-mediated neuroinflammation (Fig. 9b).

In addition to the essential role of APN in maintaining physiological functions of the CNS, there are increasing amount of evidence supporting the neuroprotective role of APN in the neurodegenerative conditions [22, 48–54]. We and other research groups have developed therapeutic strategies to target activation of APN signalling for AD treatment. Our group has demonstrated that chronic oral administration of adipoRon can increase cerebral insulin sensitivity for AD treatment [19]. Moreover, adipoRon has also been shown to induce autophagy to clear amyloid in AD mice through AMPK signaling [55] and can modulate microglia activities in the brains of mouse models [19, 56]. In addition, a recent study reported that AdipoRon mitigated tau pathology and restored mitochondrial dynamics via AMPK-related pathway in P301S tau transgenic mouse model [65]. Osmotin, the plant homolog of APN, can also reduce amyloid deposition by activating the non-amyloidogenic pathway [57]. This peptide has also shown suppressive effects on neuroinflammation [58]. Apart from the CNS benefits, APN can enhance peripheral insulin sensitivity, inhibit inflammation and oxidative stress. Since obesity, diabetes, and cardiovascular diseases are the risk factors for AD and reduction of circulatory APN is common in these conditions, the liver-specific AAV-APN^Tri^ strategy may help to protect these patients from getting AD. In addition, systemic inflammation in a secondary phenotypes of Alzheimer’s disease, the overexpressed APN^Tri^ may also provide benefits to the periphery inflammation. Further investigation is needed to validate the use of AAV-APN^Tri^ as protective treatment, early intervention or periphery actions that may also lead to the CNS improvement. Nonetheless, the adiponectin paradox has also been proposed in AD patients. It is hypothesized that AD patients may have adiponectin resistance. There is a discussion about activating APN signaling may be harmful to non-dementia disorders since prospective studies have indicated high APN levels positively correlated to all-cause and cardiovascular mortality [59]. Several groups have also reported that increased serum APN is associated with the risk of AD. Nonetheless, there is no cause-to-effect study showing high APN level leads to disease pathogenesis. However, we and other groups have reported that AD patients have reduced cerebral APN levels, particularly the low molecular APN. Importantly, APN gene polymorphisms that are associated with the reduction of adiponectin levels are also associated with an increased risk of AD [60, 61]. Our mouse model *APN*^*−/−*^5xFAD has demonstrated that APN deficiency manifests AD pathology and cognitive impairments in AD [19]. Consistently, a recent study has shown that adiponectin deficiency accelerated brain aging via mitochondria-associated neuroinflammation [62]. In contrast, several publications demonstrate an increase of APN level in mice improves the health and lifespan. It has been shown that the administration of APN receptor agonist, adipoRon, increased the lifespan of both normal and diabetic mouse models [63]. More importantly, a recent article has demonstrated overexpression of APN in mice reduces fat accumulation and prevents early mortality [64]. These reports revealed that high APN level and activation of APN signalling improve metabolic syndrome, decreases mortality, and increase lifespan.

In summary, the present study shows that APN deficiency in AD mice leads to severe microglial NLRP3 inflammasome activation. Overexpressing trimeric APN, which can cross the BBB and suppress microglial-mediated neuroinflammation and improve memory functions in AD mice. We are the first group demonstrating periphery overexpression of APN by AAV can suppress microglial NLRP3-inflammasome activation that is a potential treatment strategy for neurodegeneration for AD.

## Methods

### Plasmid construction and AAV2/8 generation

Mouse adiponectin cDNA with FLAG epitope (Origene, Catalog #MR227515) was subcloned into adeno-associated viral (AAV) vector under control of apolipoprotein E (Apo E) enhancer and human alpha-1 antitrysin (hAAT) promoter, followed by production, purification and titrated as previously described [29]. Mutation in mouse adiponectin gene C39S (cysteine to serine) was created by site directed mutagenesis kit (Agilent, Catalog #200518) to ensure that only trimeric APN was produced by the transduced cells. The oligos used to generate the C39S mutation were designed as described previously [66]. A Flag-tag sequence was introduced to the 3’end of APN^C39S^ mRNA and subcloned to the pAAV2/8 plasmid.

Vector construction: (AAV2/8-eGFP), a gift from Dr. Ian Alexander. In brief, the 734-base pair coding sequence of eGFP, with optimized Kozak sequence, was inserted as an EcoRI/EcoRV fragment under the transcriptional control of the hAAT promoter (–347 to + 56) downstream of two copies of the 319-base pair hepatic control region of human ApoE. Within this promotor, the AAV vector only transduced liver cells. The AAV2/8-APN^C39S^-Flag was generated by replacing eGFP sequence with APN^C39S^-Flag sequence. Both vectors were pseudo- serotyped with AAV8 capsid using p5E18-VD2/8.

Vector production, purification, and titration: The vectors were packaged by triple transfection of HEK293 cells with either pAAV2/8-eGFP or pAAV2/8-APN^Tri^-Flag, p5E18-VD2/8, and adenoviral helper plasmid pXX6 by calcium phosphate/DNA co-precipitation. Vector particles were purified from cell lysate by polyethaleneglycol-ammonium sulphate method and dialysed as described previously [67]. Vector genomes were titered using real-time qPCR. Primer and probe sequences specific for the bovine growth hormone polyadenylation signal region are 5’-TCTGAGTAGGTGTCATTCTATTCTGGG-3’ (sense), 5’-ACGTAGCCATGCTCTAGTCG-3’ (antisense) (both at 0.9 μmol/L), and probe 5’- TCTTCCCAATCCTCCCCCTTGCTGTC-3’ FAM/BHQ (Biosearch Technologies, Novato, CA) (0.25μol/L). Reactions were performed with AmpliTaq Gold DNA polymerase (ThermoFisher Scientific, Catalog #4398813). Amplification conditions were 95 °C for 10 min followed by 40 cycles of 95 °C for 20 s and 60 °C for 60 s. Quantitative-PCR was performed on a HD7900 Fast Real-Time PCR system (ThermoFisher Scientific). Titering of the viral particles were determined by standard curve using pAAV2/8-APN^Tri^-Flag as the template.

### Transgenic mouse model of AD and AAV-APN gene therapy

Male transgenic mice (5XFAD) carrying overexpress mutant human APP(695) with Swedish (K670N, M671L), Florida (I716V) and London (V717I) Familial Alzheimer’s disease (FAD) mutations along with human PS1 harboring two FAD mutations, M146L and L286V was used as animal model of AD [68]. 5xFAD transgenic mice were crossed to *APN*^*−/−*^ mice to generate *APN*^*−/−*^5xFAD mice. Genotyping was performed by polymerase chain reaction (PCR) of tail DNA as described previously. 4-month-old male 5xFAD mice were received AAV-APN gene therapy via intravenous injection (via tail vein) with purified AAV at a dosage of 1X10^11^ vector genomes, 100 μl of vector or phosphate-buffered saline was injected using a 33-gauge needle and syringe (Hamilton, Reno, NV) (n = 11–12 in each group). Body weight was monitor weekly. The final body weight and liver weight were determined after the mice were sacrificed. All mice (4–5 mice per cage) were maintained until 9 months old with standard conditions (23 ± 2 °C, 60–70% relative humidity, 12 h light/dark cycle,) provided with free access to food and water in the Laboratory Animal Unit of the University of Hong Kong. All animal studies were approved by the Committee on the Use of Live Animals in Teaching and Research of the University of Hong Kong.

### Quantification of Vector copy number after AAV2/8-APN^Tri^ administration

Genomic DNA was extracted from the tissues collected from mice administered with different doses of AAV2/8-APN^Tri^ particles. Vector copy number was quantified using a quantitative real-time qPCR assay with the same primers and probes used in the titering experiment mentioned above. Circular pAAV2/8-APN^Tri^ plasmid was used to generate a standard curve and quantify more accurately the rAAV genome in different tissues.

### Cell culture

Mouse microglia BV2 cells were a generous gift from Dr. Nicolai Savaskan (Universitätsklinikum Erlangen (UKER), Germany. BV2 cells were cultured in Dulbecco’s modified Eagle’s medium (DMEM) (Gibco, Catalog #19965092) with 10% fetal bovine serum (FBS) (Invitrogen, USA) and 1% penicillin/streptomycin (Gibco, Catalog #15140122). The cells were grown in a humidified incubator at 37 °C with 5% CO_2_. BV2 cells were pre-treated with mammalian trimeric APN (10 μg/ml) (Immunodiagnostics, Catalog #42,013) for 3 h and then treated with AβO (10 μM) for 24 h in serum-free culture medium. Cell lysate was collected for immunoblotting and culture medium was collected for ELISA analysis.

### Aβ Oligomers (AβO) preparation

AβO were prepared as described previously [9]. In brief, 1 mg of Aβ_42_ peptide (Abcam, Catalog #ab120301) was dissolved in 221.7 μl cold HFIP (1,1,1,3,3,3-hexafluoro-2-propanol) (Sigma-Aldrich, Catalog #105,228) to a concentration of 1 mM. The solution was then incubated at room temperature (RT) for 1 h, and placed on ice for 10 min. The solution was aliquoted into non-siliconized microcentrifuge tubes (100 μl solution containing 0.45 mg Aβ_42_) and then dried overnight at RT. The residues were dissolved in 20 μl dimethyl sulfoxide (DMSO) and then added with F12 medium to obtain a 100 μM stock solution. The solution was incubated at 4 °C overnight and then centrifuged at 14,000 × *g* for 10 min at 4 °C. Then, the Aβ oligomers were presented in the supernatant. The presence of Aβ oligomers was confirmed by immunoblot using anti-Aβ 4G8 antibody (1:1000, BioLegend, Catalog #800710).

### Behavioral tests

#### Morris water maze

Spatial memory was evaluated in 8-month-old wildtype (*n* = 9), AAV-eGFP-treated 5XFAD (*n* = 11), and AAV-APN^Tri^-treated 5XFAD (*n* = 11) female mice with the MWM paradigm. The water maze included a 90-cm diameter pool filled with opaque water (21–22 °C). A platform was submerged 0.5 cm below the water surface in the centre of one of the pool quadrants. Visible platform training was performed in 2 consecutive days (4 trials/sessions; 2 sessions/day). The hidden platform remained at the same position throughout the trials. Training consisted of one session (4 trials/session) for 5 consecutive days. Each trial ended when the animal reached the platform. The animal had a maximum of 60-s to reach the platform, after which it was manually guided to the platform. Once the mice get onto the platform, the animal was given a 60-s rest before being returned to its cage. The inter-trial interval was ∼1 h. On the 8th training session, a probe test was performed. In this memory retention test, the platform was removed, and the mouse was allowed to navigate for 60-s. The path of each mouse and the time spend in each quadrant were recorded. After the test, mice were then sacrificed, and the brains were collected for further biochemical and pathological analyses.

#### Samples collection

For immunohistochemistry, mice were perfused transcardially with PBS and then with ice-cold 4% paraformaldehyde in PBS. Brains were removed and fixed in 4% paraformaldehyde at 4 °C for at 48 h. The brains were serially dehydrated in increasing ethanol gradients and embedded in wax. Paraffin sections were prepared at 5 µm thickness and mounted onto SuperFrost Plus slides.

For immunoblotting, mice were perfused transcardially with PBS and brain will be rapidly removed and put in liquid nitrogen, then stored in -70 °C until use. Brain homogenates were prepared as described previously [22]. Protein concentration of each lysates was determined by BCA assay following the manufacturer protocol (Biorad, Catalog # 5000111).

#### Immunohistochemistry and Immunofluorescence staining

Brain tissues (sectioned in 10μm) were studied for immunohistochemistry of Aβ with antibodies against Aβ residues 17–24 clone 4G8 (BioLegend, Catalog #800710), followed by HRP-anti-mouse secondary antibody (Dako, P0447) at RT for 30 min. Staining was then developed by 3,3′-diaminobenzidine liquid substrate (Dako, Catalog #K3468). Then, slides were counterstained with hematoxylin and mounted with Permount. Images were acquired with a Leica DM1000 LED microscope with microscope camera ICC 50W (Leica Microsystems, Wetzlar, Germany).

For immunofluorescence staining, microglia activation was detected by rabbit anti-Iba1 antibody (1:100, Wako Chemical, Catalog #019–19741), and Goat anti-Iba1 antibody (1:100, Novus Biologicals, Catalog #NB100-1028), coimmunostained with mouse-anti-NLRP3 (1:200, adipogen, Catalog #AG-20B-0014), and rabbit anti-ASC (1:1000, Cell Signaling Tech. Catalog #67,824), astrocytic activation with GFAP antibody (Santa Cruz Biotech., Catalog #sc-58766), plaque-associated dystrophic neurites by rabbit anti-Lamp1 antibody (1:500, Abcam, Catalog #ab208943) followed by appropriate Alexa-Fluor-conjugated secondary antibody (Thermo Fisher Scientific) at RT for 1 h and thioflavin S staining as described previously [19]. Sections were mounted with slow fade® anti-fade reagent with or without DAPI (Thermo Fisher Scientific, P36931). Images acquisition was done by Nikon EclipsHe NiU microscope (Nikon Instruments Inc., Melville, USA). Same exposure setting was used across each experiment groups. For double-immunofluorescence staining, images were merged in the software Nikon NIS-Element (Nikon Instruments Inc., Melville, USA) and exported as image files for further quantification analysis by ImageJ software (Wayne Rashband NIH, USA).

#### Image analysis

To quantify Aβ-associated dystrophic neurites, confocal z-stack images of different hippocampi regions were acquired. Image acquisition was performed by Zeiss LSM710 confocal laser scanning microscope in the Centre of PanorOmics Sciences at the LKS Faculty of Medicine the University of Hong Kong. Images were taken using the 20X air 0.75 NA as Z stacks of 0.5 µm/step and exported to Imaris (Bitplane 9.5.3) and Image J (NIH) for analysis. To determine the thioflavin S-positive plaque, z-stack images were scanned and judge manually. Using *z*-stack maximum intensity projected images, the diameters of thioflavin-S^+^amyloid plaques were automatically calculated by ‘particle analysis’ following ‘threshold’ Plug-in in Fiji software (NIH). Compact plaques with diameter upto 6μm and filamentous Aβ were selected for further plaque-associated dystrophic neurites analysis. To measure dystrophic neurites, z-projection from the centre of each plaque core was done by ImageJ. The Lamp1-stained area representing dystrophic neurites was measured by ImageJ. The Lamp1-stained area was then converted to volume to estimate the volume of dystrophic neurites.

### Immunoblotting and Enzyme Linked Immunosorbent Assay (ELISA)

Tissues lysates or cell lysates were studied by standard SDS-PAGE and western blotting for detecting mouse-anti-NLRP3 (1:200, adipogen, #AG-20B-0014), rabbit anti-ASC antibody (1:1000, adipogen, Catalog #AL177), rabbit anti-ASC (1:1000, Cell Signaling Tech. Inc, Catalog #6,824), rabbit anti-caspase-1 (1:1000, Cell Signaling Tech. Inc, Catalog #83383), rabbit anti-synaptophysin (1:1000, abcam, Catalog #ab7837), rabbit anti-spinophilin (1:1000, Cell Signaling Tech. Inc, Catalog #14136), rabbit-anti-flag (1:1000, Sigma Aldrich, Catalog #14793), and mouse-anti-Adiponectin (ThermoScientific, Catalog #MA1-054) were incubated at 4 °C overnight, followed by HRP-conjugated secondary antibodies (goat anti-rabbit, 1:5000 or rabbit anti-mouse, 1:5000; Dako, Glostrup, Denmark) at RT for 1 h. Loading control was performed in each experiment by HRP-conjugated anti-α-Tubulin (1:1000, Cell Signaling Tech. Inc, Catalog #2125). Every experiment has the loading control detection unless the molecular size of the target protein has similar molecular size with the α-Tubulin. A loading control was then run in a separated gel in the same experiment. The immunoblot signals were visualized by Westernbright Quantum HRP substrate (advansta, Catalog #490005-06) and then detected by ChemiDoc Imaging system (Bio-Rad, USA).

Amyloid-β_40_ and amyloid-β_42_ in the soluble brain lysates were measured using the human Aβ(140) and human Aβ(1–42) ELISA Kit (Wako, Catalog #292-62301 and Catalog #296-64401). IL-1β and IL-18 levels in the brain lysates were measured using the mouse IL-1β ELISA kit (RayBiotech Life, Catalog #ELM-IL1b-1) and the mouse IL-18 ELISA kit (RayBiotech Life Inc., Catalog #ELM-IL18BPC). The assays were performed following supplier instructions. The optical density of each well at 450 nm was determined by a CLARIO star microplate reader (BMG LABTECH, Germany).

### Statistical analysis

All data are expressed as the mean ± SEM. Statistical analyses were performed with GraphPad Prism 6 (GraphPad Software). For behavioral experiments, MWM data was analysed with two-way ANOVA followed by Bonferroni’s post hoc test. Other behavioural tests were analysed with one-way ANOVA, followed by Bonferroni’s post hoc test. In other experiments, between-group differences were determined with one-way ANOVA, followed by Bonferroni’s post hoc test. Alternatively, the mean significant difference between two groups was determined with two-tailed unpaired Student’s *t*-test. Each figure legend specifies the statistical test used. Statistical significance was defined as: *p < 0.05, **p < 0.01, and ***p < 0.001.

### Supplementary Information

**Additional file 1: Figure S1.** Adiponectin deficiency exacerbates microgliosis in the early adult stage but not in aged AD mice.** A** Immunofluorescent staining of Iba1 indicates the changes of spatiotemporal microgliosis in the hippocampus of wildtype, *APN*^*−/−*^, 5xFAD, and *APN*^*−/−*^5xFAD mice at different age (3, 6, 9, 12, 15, 18-month) (n = 3 mice for each experimental group) (Magnification 10 × 20; Scale bar: 50μm). **(B)** Relative intensity of microglial Iba1 staining in the hippocampus of wildtype, *APN*^*−/−*^, 5xFAD, and *APN*^*−/−*^5xFAD mice at different age (3, 6, 9, 12, 15, 18-month). Data were presented as the mean ± s.e.m. n.s. not significant; *p < 0.05; **p < 0.01; ***p < 0.001. Statistical analysis was performed by one-way ANOVA followed with Bonferroni’s post hoc comparison tests. **Figure S2.** No mononuclear cell infiltration into the liver after AAV administration. H & E staining of the liver in wildtype, AAV2/8-GFP-treated, and AAV2/8-APN^Tri^-treated 5xFAD indicated no infiltration of immune cell in the liver of AAV-treated mice. (n = 4 mice for each experimental group) (Magnification: 10 × 10; Scale bar: 50μm). **Figure S3. **AAV2/8-APN^Tri^ treatment exerts no significant effect in motor functions and increases level of synaptic proteins in the brain of AD mice.** A** Total distance travelled of wildtype, AAV2/8-GFP-treated and AAV2/8-APN^Tri^-treated 5xFAD in the visible platform of Morris Water Maze. (n = 9 mice for wildtype and n = 11 for both AAV-treated groups). **B** Average swim speed of wildtype, AAV2/8-GFP-treated and AAV2/8-APN^Tri^-treated 5xFAD in the visible platform of Morris Water Maze. (n = 9 mice for wildtype and n = 11 for both AAV-treated groups). **C** Western blotting assay of synaptophysin and spinophilin in the hippocampus of AAV2/8-GFP-treated and AAV2/8-APN^Tri^-treated 5xFAD. (n = 5 mice for each experimental group). **D** Densitometric analysis of synaptophysin and spinophilin in the hippocampus of AAV2/8-GFP-treated and AAV2/8-APN^Tri^-treated 5xFAD. Data were presented as the mean ± s.e.m. n.s. not significant; *p < 0.05; **p < 0.01; ***p < 0.001. Statistical analysis (A, B) were performed by one-way ANOVA followed with Bonferroni’s post hoc comparison tests and (D) was performed by unpaired t test. **Figure S4. **AAV2/8-APN^Tri^-treated reduces fibrillar amyloid deposition in the cortex and astrogliosis in the cortex and hippocampus of 5xFAD mice.** A** Thioflavin staining of Aβ (green) in the cortex of AAV2/8-GFP-treated (n = 6), and AAV2/8-APN^Tri^-treated 5xFAD mice (n = 6). (Magnification: 10 × 20; Scale bar: 50μm). **B** Relative intensity of the thioflavin-stained Aβ cortex of AAV2/8-GFP-treated, and AAV2/8-APN^Tri^-treated 5xFAD mice. **C** GFAP (red) and thioflavin S (green) double-immunofluorescent staining in the hippocampus and cortex of AAV2/8-GFP-treated (n = 6), and AAV2/8-APN^Tri^-treated 5xFAD mice (n = 6). (Scale bar: 50μm). **D** Relative intensity of GFAP in the hippocampus and cortex of AAV2/8-GFP-treated, and AAV2/8-APN^Tri^-treated 5xFAD mice. Data were presented as the mean ± s.e.m. n.s. not significant; *p < 0.05; **p < 0.01; ***p < 0.001. Statistical analyses were performed by unpaired t test. **Table S1. **Recombinant AAV2/8 did not cause significant changes of body weight, weight changes, and liver weight 20 weeks after administration. Wildtype (n = 9), 5xFAD (n = 7), AAV2/8-eGFP treated 5xFAD (n = 11), and AAV2/8-APN^Tri^ treated 5xFAD (n = 11).

## Data Availability

Not applicable.

## Declarations

### Study approval

All experimental procedures were approved by the Committee on the Use of Live Animals in Teacher and Research (CULATR No: 3864-16) at the University of Hong Kong.
