# Supplementary material for: Liver-specific adiponectin gene therapy suppresses microglial NLRP3-inflammasome activation for treating Alzheimer’s disease
Source: J Neuroinflammation. 2024 Mar 27;21:77. doi: 10.1186/s12974-024-03066-y (PMC10967198; doi:10.1186/s12974-024-03066-y)
Supplement: Supplementary file 1 — Additional file 1: Figure S1. Adiponectin deficiency exacerbates microgliosis in the early adult stage but not in aged AD mice. A Immunofluorescent staining of Iba1 indicates the changes of spatiotemporal microgliosis in the hippocampus of wildtype, APN−/−, 5xFAD, and APN−/−5xFAD mice at different age (3, 6, 9, 12, 15, 18-month) (n = 3 mice for each experimental group) (Magnification 10 × 20; Scale bar: 50μm). (B) Relative intensity of microglial Iba1 staining in the hippocampus of wildtype, APN−/−, 5xFAD, and APN−/−5xFAD mice at different age (3, 6, 9, 12, 15, 18-month). Data were presented as the mean ± s.e.m. n.s. not significant; *p < 0.05; **p < 0.01; ***p < 0.001. Statistical analysis was performed by one-way ANOVA followed with Bonferroni’s post hoc comparison tests. Figure S2. No mononuclear cell infiltration into the liver after AAV administration. H & E staining of the liver in wildtype, AAV2/8-GFP-treated, and AAV2/8-APNTri-treated 5xFAD indicated no infiltration of immune cell in the liver of AAV-treated mice. (n = 4 mice for each experimental group) (Magnification: 10 × 10; Scale bar: 50μm). Figure S3. AAV2/8-APNTri treatment exerts no significant effect in motor functions and increases level of synaptic proteins in the brain of AD mice. A Total distance travelled of wildtype, AAV2/8-GFP-treated and AAV2/8-APNTri-treated 5xFAD in the visible platform of Morris Water Maze. (n = 9 mice for wildtype and n = 11 for both AAV-treated groups). B Average swim speed of wildtype, AAV2/8-GFP-treated and AAV2/8-APNTri-treated 5xFAD in the visible platform of Morris Water Maze. (n = 9 mice for wildtype and n = 11 for both AAV-treated groups). C Western blotting assay of synaptophysin and spinophilin in the hippocampus of AAV2/8-GFP-treated and AAV2/8-APNTri-treated 5xFAD. (n = 5 mice for each experimental group). D Densitometric analysis of synaptophysin and spinophilin in the hippocampus of AAV2/8-GFP-treated and AAV2/8-APNTri-treated 5xFAD. Data were presented as [file 12974_2024_3066_MOESM1_ESM.pdf]

Supplementary Figure 1

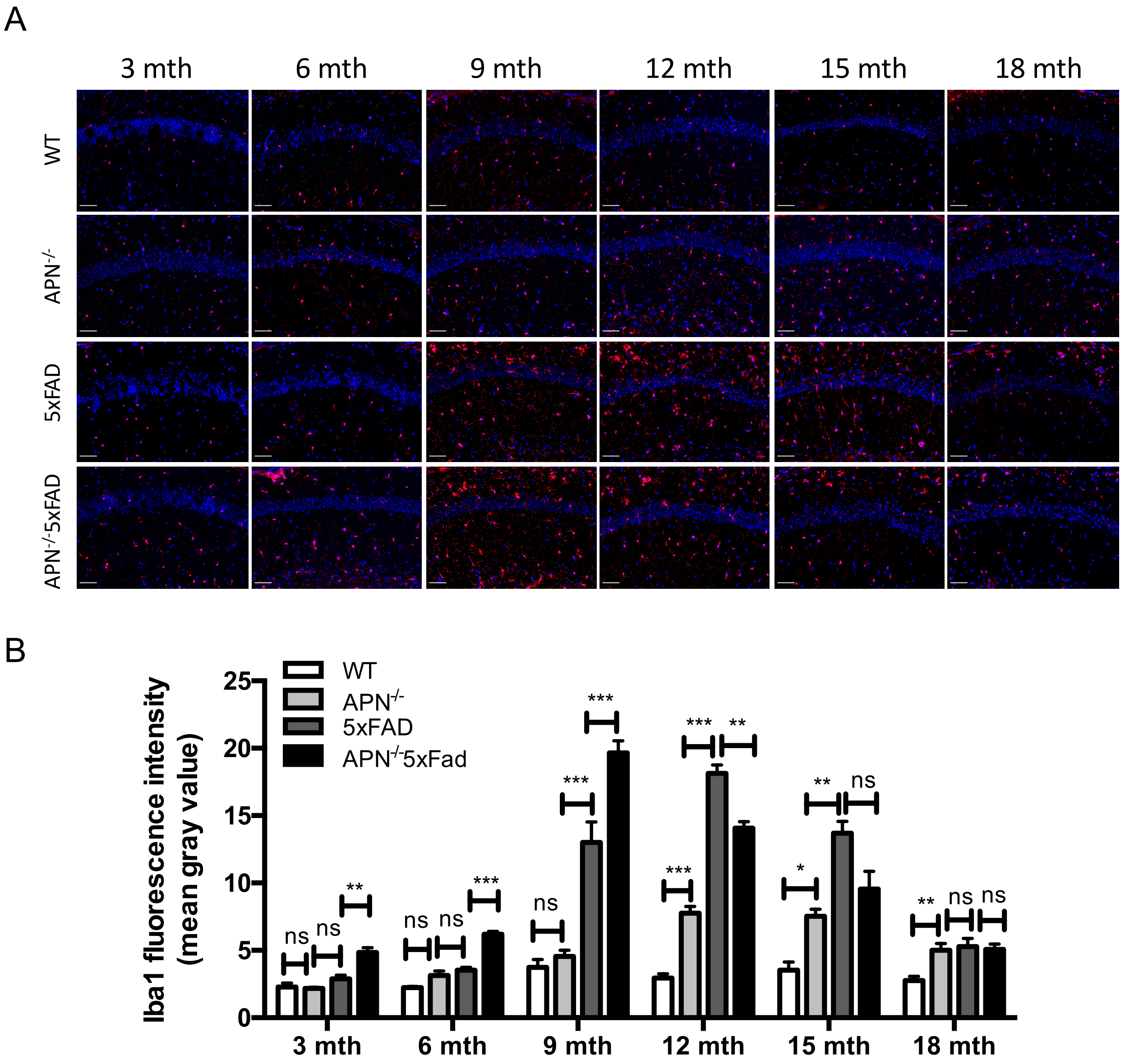

Supplementary Figure 2

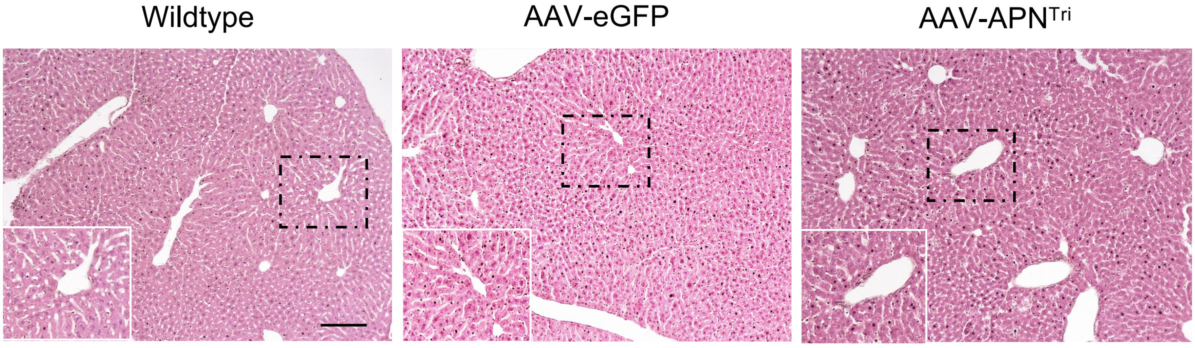

Supplementary Figure 3

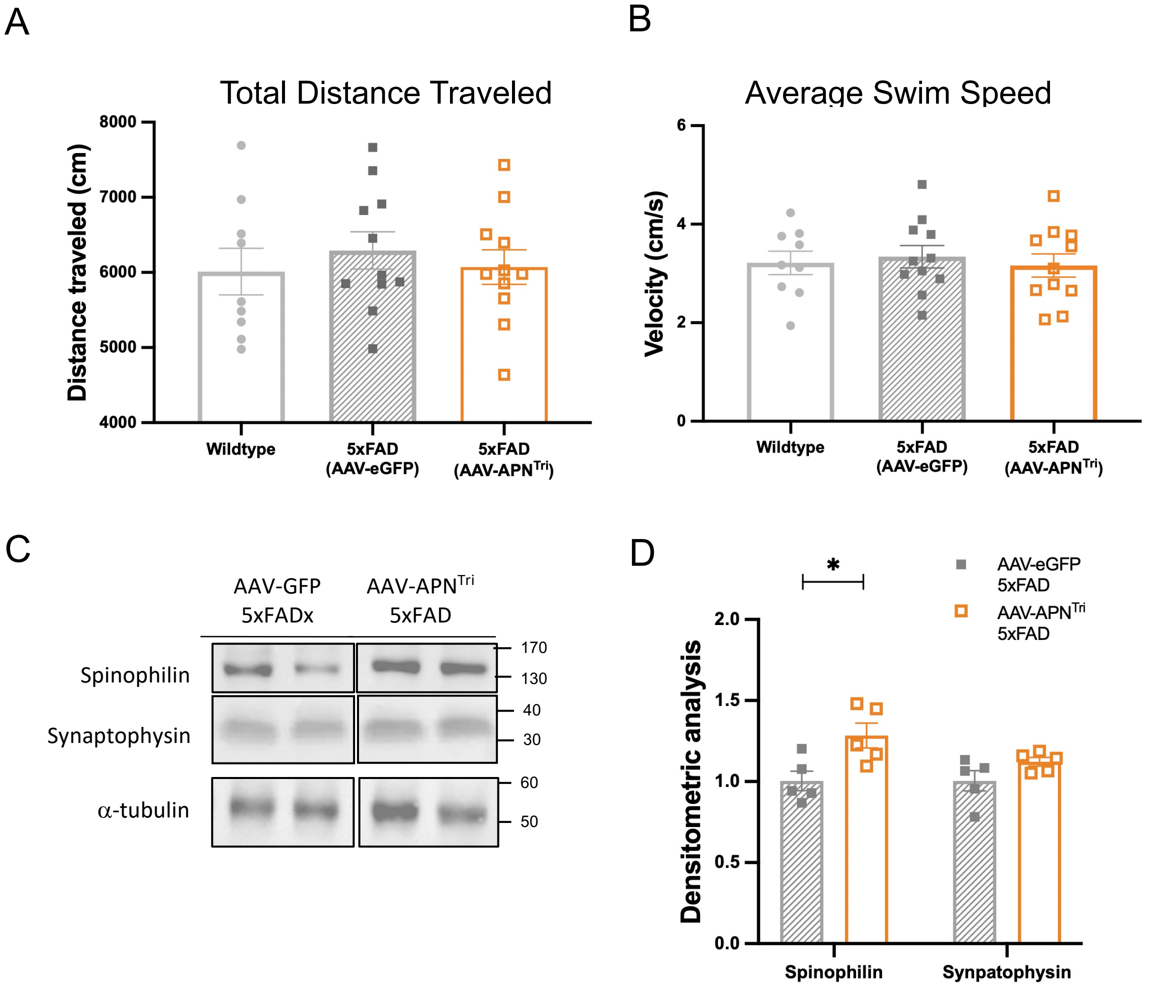

Supplementary Figure 4

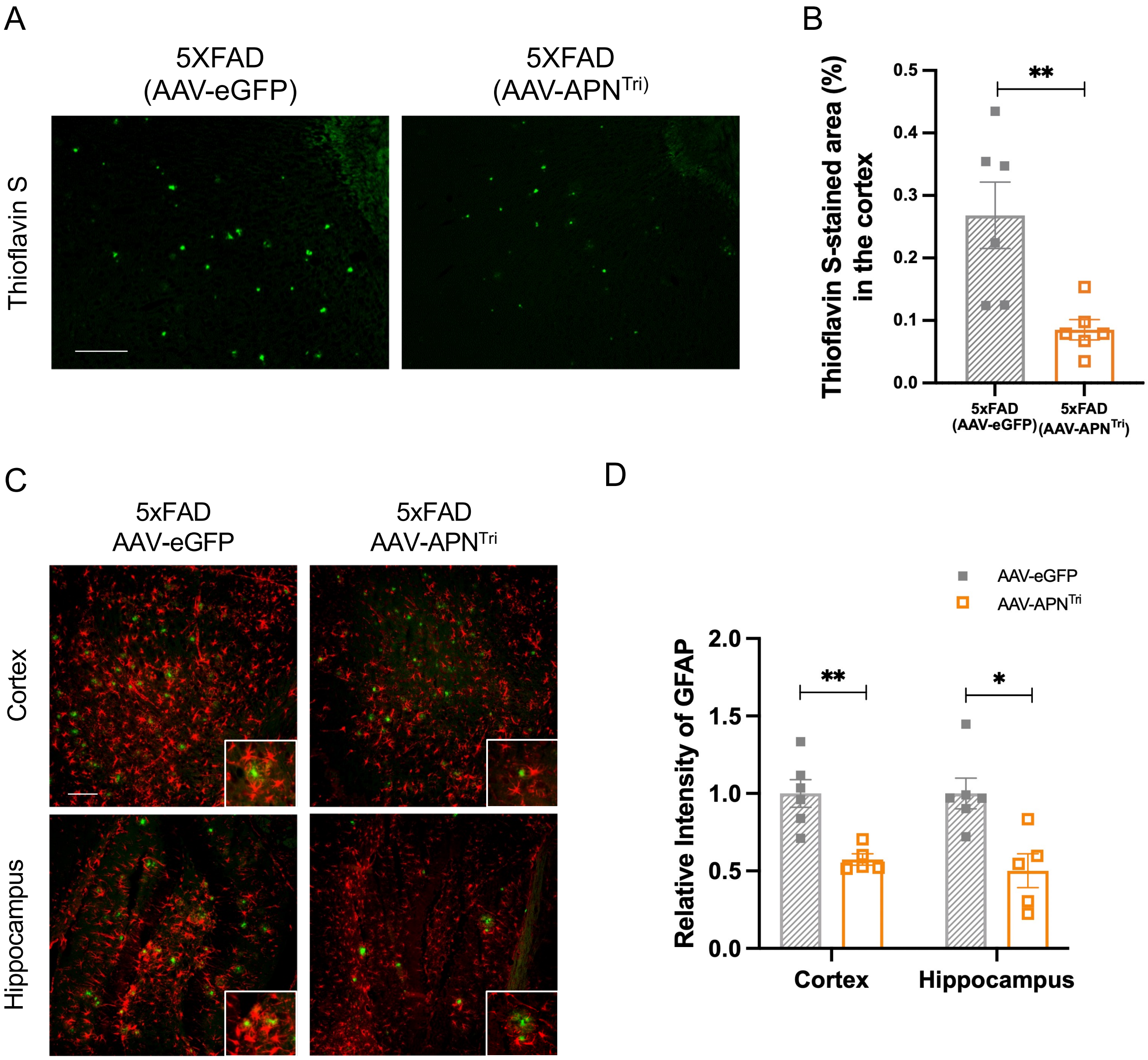

Supplementary Table 1. Body Weights, Weight Changes and Liver Weights 20 weeks after AAV2/8 administration

	Wildtype	5xFAD	5xFAD (AAV-eGFP)	5xFAD (AAV-APN^{Tri})
Weight (g)	30.600 ± 0.675	28.541 ± 0.799	28.490 ± 0.377	29.403 ± 0.797
Weight Change (g)	1.85 ± 0.3252	0.9075 ± 0.4844	0.7573 ± 0.3975	0.9522 ± 0.1407
Liver (g)	1.720 ± 0.025	1.600 ± 0.384	1.657 ± 0.020	1.682 ± 0.019
Liver weight / Body Weight (mg/g)	59.96 ± 1.07	56.16 ± 1.06	58.25 ± 0.89	57.49 ± 1.14
